# Disruption of metabolic licensing by JAK inhibitors constrains CD8 T cell activation and effector function

**DOI:** 10.1038/s41419-026-08610-7

**Published:** 2026-03-24

**Authors:** Luisina Inés Onofrio, Carolina Abrate, Ingrid Strusberg, Liliana Morales, Danilo Guillermo Ceschin, Carolina Lucía Montes, Cinthia Carolina Stempin, Eva Acosta Rodríguez

**Affiliations:** 1 Centro de Investigaciones en Bioquímica Clínica e Inmunología (CIBICI-CONICET), Facultad de Ciencias Químicas, Universidad Nacional de Córdoba (UNC), Córdoba, Argentina; 2 Instituto Médico Strusberg, Córdoba, Argentina; 3 Centro de Investigación en Medicina Translacional “Severo R. Amuchástegui”, Consejo Nacional de Investigaciones Científicas y Técnicas, (CIMETSA-CONICET), Córdoba, Argentina

**Keywords:** CD8-positive T cells, Autoimmunity, Rheumatoid arthritis

## Abstract

Janus kinase inhibitors (JAKis) are widely prescribed for autoimmune diseases, but their use is associated with increased infection risk. The mechanisms underlying this susceptibility remain unclear. CD8 T cells play a central role in antimicrobial defence, yet little is known about how JAKis reprogramme their activation and effector programmes. Here, we investigated naive and memory CD8 T cells from healthy donors stimulated in vitro in the presence of clinically relevant JAK inhibitors targeting JAK1 (JAK1i), JAK1/2 (JAK1/2i), or JAK1/3 (JAK1/3i). Flow cytometry, SCENITH, transmission electron microscopy, and RNA-seq were used to evaluate metabolic and functional programmes. We found that JAKis uncoupled phenotypic activation from metabolic reprogramming. Functionally, JAKi-treated CD8 T cells exhibited reduced activation and produced lower amounts of cytokines and cytotoxic molecules. Notably, even JAKi-treated memory CD8 T cells that upregulated CD69 and CD25 failed to engage glycolysis, showing decreased GLUT1 expression and glucose uptake. SCENITH profiling confirmed diminished glucose dependence and a shift toward mitochondrial reliance, despite reduced mitochondrial potential and structural alterations. Transcriptomic and protein analyses further revealed decreased mTOR activity and increased p53-associated transcripts, consistent with impaired growth and stress signalling. CD8 T cells from rheumatoid arthritis patients under JAKi therapy were analysed ex vivo for translational validation. These cells showed similar metabolic and signalling alterations, underscoring their clinical relevance. Altogether, these findings identify JAKis as disruptors of metabolic and signalling pathways in CD8 T cells, providing a mechanistic link between impaired effector function and the increased infection risk observed in treated patients.

## Introduction

Janus kinase (JAK) inhibitors (JAKis) are small-molecule drugs that block cytokine signalling through the JAK/STAT pathway, modulating inflammatory responses [1]. These agents have rapidly expanded the therapeutic landscape, providing effective treatments across multiple immune-mediated diseases, including rheumatoid arthritis (RA), psoriatic arthritis, ulcerative colitis, atopic dermatitis, and alopecia areata [2–5]. In RA, a chronic systemic disorder characterised by synovial inflammation, joint damage, and functional impairment, JAKis have transformed disease management, achieving disease control in patients refractory to conventional or biologic disease-modifying antirheumatic drugs (DMARDs) [6]. Currently approved JAKis differ in their selectivity for individual JAK family members, with tofacitinib primarily targeting JAK1 and JAK3, baricitinib acting on JAK1 and JAK2, and upadacitinib showing high selectivity for JAK1. This pharmacological diversity underscores that distinct JAKis may differentially modulate cytokine signalling pathways [7–9].

Despite these clinical successes, safety concerns have emerged. Recent studies in JAKis-treated patients report a higher incidence of herpes zoster reactivation and opportunistic infections like tuberculosis [10–12]. These findings raise critical questions about the impact of JAK inhibition on cellular immunity, which is crucial for the control of intracellular pathogens. Among its components, CD8 T cells are central effectors, with a pivotal role in antiviral defence through their capacity to eliminate infected cells and secrete proinflammatory cytokines [13].

Upon activation, both naive and memory CD8 T cells undergo clonal expansion and acquire effector functions, processes sustained by a metabolic reprogramming that supports their increased biosynthetic and energetic demands [13]. Resting T cells remain metabolically quiescent, relying primarily on oxidative phosphorylation (OXPHOS) for energy production. In contrast, activation triggers a metabolic shift characterised by aerobic glycolysis, enhanced amino acid uptake, and mitochondrial remodelling [14, 15]. These adaptations are orchestrated by signalling pathways such as mTOR and MYC, which integrate antigenic and cytokine inputs with nutrient availability to drive proliferation and effector differentiation [16–18].

Although cytokine-driven JAK/STAT signalling has been implicated in the regulation of T-cell metabolic pathways [19], the direct consequences of pharmacologic JAK inhibition on T-cell metabolism and effector programmes remain poorly defined. Previous work, including our own, has shown that tofacitinib can promote immunosenescence and impair effector activity in T cells [20, 21]. However, it remains uncertain whether these effects are unique to tofacitinib, a JAK1/3 inhibitor, or extend to other JAKis, and, importantly, how such drugs interfere with the metabolic programmes that sustain CD8 T-cell responses. Because metabolic pathways not only fuel effector differentiation but also govern stress responses, senescence, and long-term T-cell fate, understanding how JAKis alter these processes is clinically relevant, particularly in light of the increased susceptibility to infections observed in treated patients.

Here, we investigated how different JAKis affect the activation, metabolism, and effector function of human CD8 T cells from PBMC of healthy donors (HD), with a focus on memory subsets given their central role in antiviral defence. We used an integrated approach that links functional and metabolic readouts with multiomic and ultrastructural analyses. Finally, we analysed PBMCs from RA patients under JAKi or methotrexate therapy to test whether these alterations occur in vivo. Together, these analyses define how JAK inhibition disrupts the cellular programmes required for effective T-cell immunity.

## Results

### JAKis impair activation and metabolic reprogramming of CD8 T cells

We previously reported that tofacitinib, a JAK1/3i, activates immunosenescence pathways while inhibiting effector functions in CD8 T cells [20]. To determine whether these effects extended to other clinically used JAKis, we compared tofacitinib with upadacitinib, a JAK1i, and baricitinib a JAK1/2i. Naïve and memory CD8 T cells from HD PBMCs were sorted (Fig. S1A) and stimulated with anti-CD3/CD28 in the presence or absence of the drugs. At 1 μM, all three inhibitors markedly reduced activation (upregulation of CD25 and CD137), proliferation (Ki-67⁺ cells) and effector responses (expression of TNF and granzyme B), with JAK1/2i showing greater potency than JAK1/3i or JAK1i, consistent with differences in JAK selectivity (Fig. S1B). Dose–response analyses confirmed that all compounds impaired activation and proliferation in a dose-dependent manner (Fig. S1C). Notably, effector molecule production was already maximally suppressed at concentrations as low as 0.1 μM, which approximates therapeutic levels in treated patients [22–24]. In addition, inhibition of TYK2, the fourth member of the JAK family, similarly reduced activation and proliferation of naive and memory CD8 T cells in a dose-dependent manner (Fig. S1D). Together, these data indicate that targeting distinct members of the JAK family converges on a potent suppression of CD8 T-cell activation and effector programming, despite differences in JAK selectivity.

To directly assess whether reduced effector molecule expression translated into impaired cytotoxic function, we performed CD8 T cell–mediated killing assays using a standardised target cell system [25]. While activated memory CD8 T cells efficiently induced target cell death across increasing effector-to-target ratios, JAK inhibition using selected inhibitors targeting JAK1/3 and JAK1/2 at the physiologically relevant concentrations markedly reduced cytotoxic activity as shown in representative experiments (Fig. S2A) and summarised across donors (Fig. S2B). These data demonstrate that JAK inhibition directly impairs CD8 T-cell cytotoxic function, reinforcing the functional relevance of the observed defects in activation and effector differentiation.

**Fig. 1:**
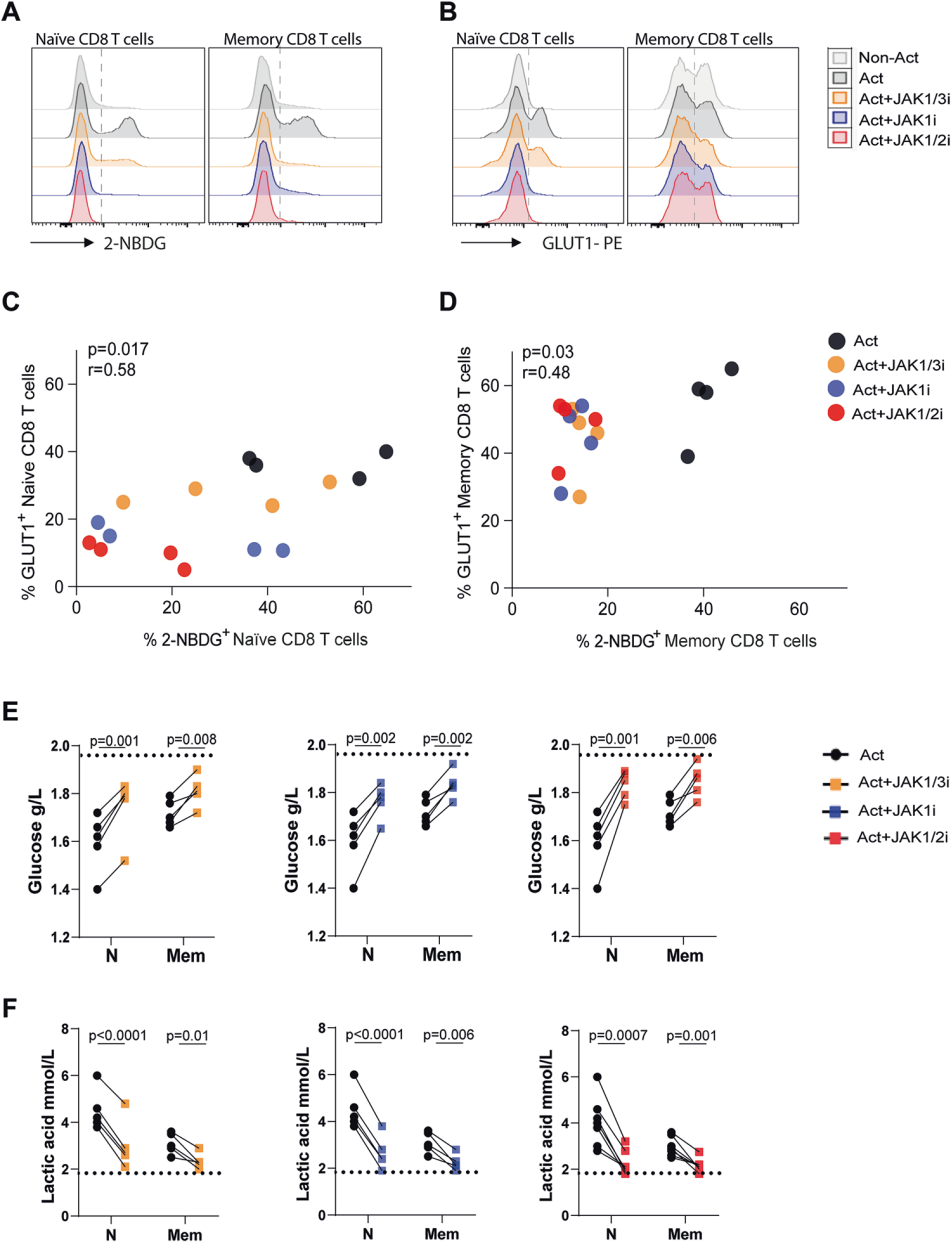
Glucose metabolism in activated CD8 T cells under JAK inhibition. Naïve and memory CD8 T cells from healthy donors (HD) were polyclonally activated (Act) for 3 days in the presence or absence of JAK1/3i, JAK1i, or JAK1/2i. Cells without stimulation were used as a control (Non-Act). **A**, **B** Representative histograms of 2-NBDG uptake and GLUT1 expression. **C**, **D** Correlation between the frequency of 2-NBDG⁺ and GLUT1⁺ cells in naive (**C**) and memory (**D**) CD8 T cells. Each bubble colour denotes a different experimental condition. Pearson correlation coefficients (r) and *P* values are indicated. **E**, **F** Lactate concentration (mmol/L) (**E**) and glucose levels (g/L) (**F**) measured in culture supernatants. Data are shown as mean ± SD from samples of 9 individual HD assayed across three independent experiments. *P* values were calculated using paired *t* test (**E**, **F**).

Because CD8 T-cell activation and effector function depend on a glycolytic shift, we next examined whether JAKis disrupt this metabolic programme. Glucose uptake, measured by 2-NBDG incorporation, was significantly reduced in both naive and memory CD8 T cells activated and treated with JAK1/3, JAK1 or JAK1/2 inhibitors compared to activated controls (Fig. 1A and Fig. S3A). This defect was associated with lower surface expression of the glucose transporter GLUT1 (Fig. 1B and Fig. S3B). A positive correlation between GLUT1 levels and the glucose analogue incorporation was observed in both CD8 T-cell subsets stimulated and treated with JAK1/3, JAK1 or JAK1/2 inhibitors (Fig. 1C, D). Consistent with impaired uptake, culture supernatants of JAKi-treated cells contained higher residual glucose and showed significantly reduced lactate secretion (Fig. 1E, F). Notably, inhibition of TYK2 at physiologically relevant concentrations similarly reduced glucose analogue incorporation and GLUT1 expression (Fig. S3C), and resulted in increased residual glucose and reduced lactate levels in culture supernatants (Fig. S3D). Together, these findings indicate a defective glycolytic flux, consistent with impaired metabolic reprogramming upon JAK inhibition.

### JAKis uncouple phenotypic activation from glycolytic reprogramming in T cells

**Fig. 2:**
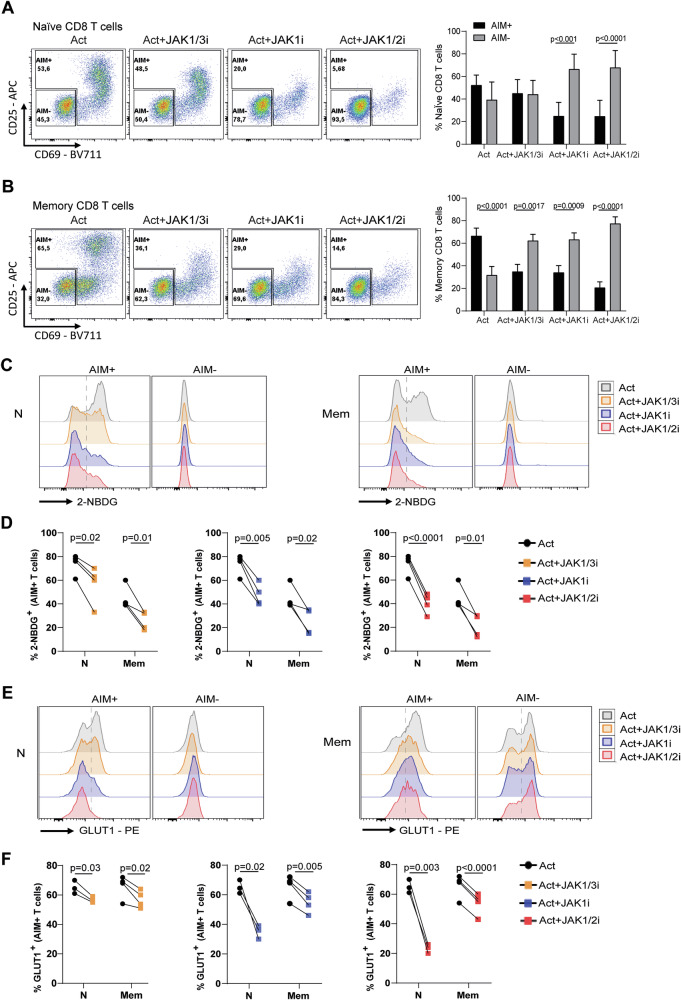
Activation markers and glucose uptake in AIM^+^ and AIM^−^ CD8 T cells under JAK inhibition. Naïve and memory CD8 T cells from healthy donors (HD) were polyclonally stimulated for 3 days in the presence or absence of JAK1/3i, JAK1i, or JAK1/2i. After culture, AIM^+^ cells were defined as those positive for at least one activation marker (CD25 or CD69), while AIM^-^ cells were negative for both markers. **A**, **B** Representative flow cytometry plots showing the frequency of AIM^+^ and AIM^-^ cells in naive (**A**) and memory (**B**) CD8 T cells under the indicated conditions (left). The right panels show the pooled data from all donors. **C**–**F** Representative histograms and pooled data of metabolic marker expression in AIM⁺ and AIM⁻ naive and memory CD8 T cells. **C** Representative histograms of 2-NBDG uptake. **D** Pooled quantification of 2-NBDG uptake in AIM⁺ naive and memory CD8 T cells. **E** Representative histograms of GLUT1 expression. **F** Pooled quantification of GLUT1 expression in AIM⁺ naive and memory CD8 T cells. Data are shown as mean ± SD from samples of 9 individual HD analysed across three independent experiments. Statistical significance in (**A**, **B**) was assessed using two-way ANOVA followed by Šídák’s multiple comparisons test. Statistical significance in (**D**, **F**) was assessed using paired *t* test.

Given that JAKis reduce overall CD8 T-cell activation and that metabolic rewiring occurs in cells that successfully engage an activation programme, we next focused on the subset of stimulated CD8 T cells expressing at least one activation-induced marker (CD25 or CD69), hereafter defined as AIM⁺ cells (Fig. 2A, B). In naive CD8 T cells, αCD3/αCD28 activation resulted in comparable proportions of AIM⁺ and AIM⁻ cells, which remained unchanged with JAK1/3i but shifted toward AIM⁻ cells with JAK1i and JAK1/2i. In contrast, memory CD8 T cells predominantly became AIM⁺ upon activation, an effect that was inverted by all JAKis, leading to reduced AIM⁺ and increased AIM⁻ frequencies. These findings indicate that JAK inhibition broadly disrupts the acquisition of an activated phenotype in both naive and memory subsets.

We then evaluated whether AIM⁺ cells exposed to JAKis were able to complete their metabolic reprogramming. Within the AIM⁺ fraction, all three inhibitors markedly reduced glucose uptake, measured by 2-NBDG incorporation (Fig. 2C, D), and decreased GLUT1 expression (Fig. 2E, F) in both naive and memory CD8 T cells. In contrast, AIM⁻ cells showed no significant changes in these parameters. These results indicate that the metabolic impairment under JAK inhibition cannot be attributed only to the reduced frequency of activated cells, but also reflects a defect in those that upregulate activation markers.

To determine whether this uncoupling between phenotypic activation and glycolytic reprogramming extended beyond CD8 T cells, we next analysed naive and memory CD4 T cells activated in vitro in the presence of a selected model JAK inhibitor targeting JAK1/2. JAK1/2 inhibition reduced the frequency of AIM⁺ CD4 T cells in both naive and memory subsets (Fig. S4A). Within activated CD4 T cells, JAK1/2 inhibition was associated with decreased glucose analogue uptake and reduced surface GLUT1 expression (Fig. S4B). Consistent with these changes, culture supernatants from JAK1/2i-treated CD4 T cells contained increased residual glucose and showed reduced lactate levels (Fig. S4C). Together, these data indicate that disruption of AIM-associated glycolytic reprogramming under JAK inhibition is not restricted to CD8 T cells.

Finally, focusing again on CD8 T cells, unsupervised clustering using FlowSOM combined with UMAP visualisation provided an integrative view of these changes. Upon stimulation, both naive and memory CD8 T cells normally upregulated a coordinated module of activation markers (CD25, CD69) together with GLUT1 and the transcription factors IRF4 and HIF-1α, key regulators of metabolic reprogramming and effector differentiation [26, 27]. This activation-associated profile was markedly reduced in JAKis-treated cells, as illustrated for JAK1/2i (Fig. S5), with similar results obtained for JAK1/3i and JAK1i (not shown). These findings confirm that JAK inhibition impairs both the acquisition of an activated phenotype and the metabolic remodelling that sustains effector differentiation.

### JAKis decrease active mitochondria and promote lipid accumulation in CD8 T cells

In addition to glucose uptake and glycolytic reprogramming, mitochondrial function is essential for T-cell activation, survival, and differentiation, as it provides biosynthetic precursors and ATP through oxidative phosphorylation (OXPHOS) [28]. In the resting state, T cells remain metabolically quiescent, relying primarily on OXPHOS and maintaining low glycolytic rates. Upon activation, the rise in mitochondrial membrane potential (Δψm) and mitochondrial reactive oxygen species (mROS), which contribute to activation and effector functions, reflects the increased metabolic demand [29]. Given that JAKis uncouple phenotypic activation from glycolytic reprogramming, we next asked whether mitochondrial fitness was also affected.

**Fig. 3:**
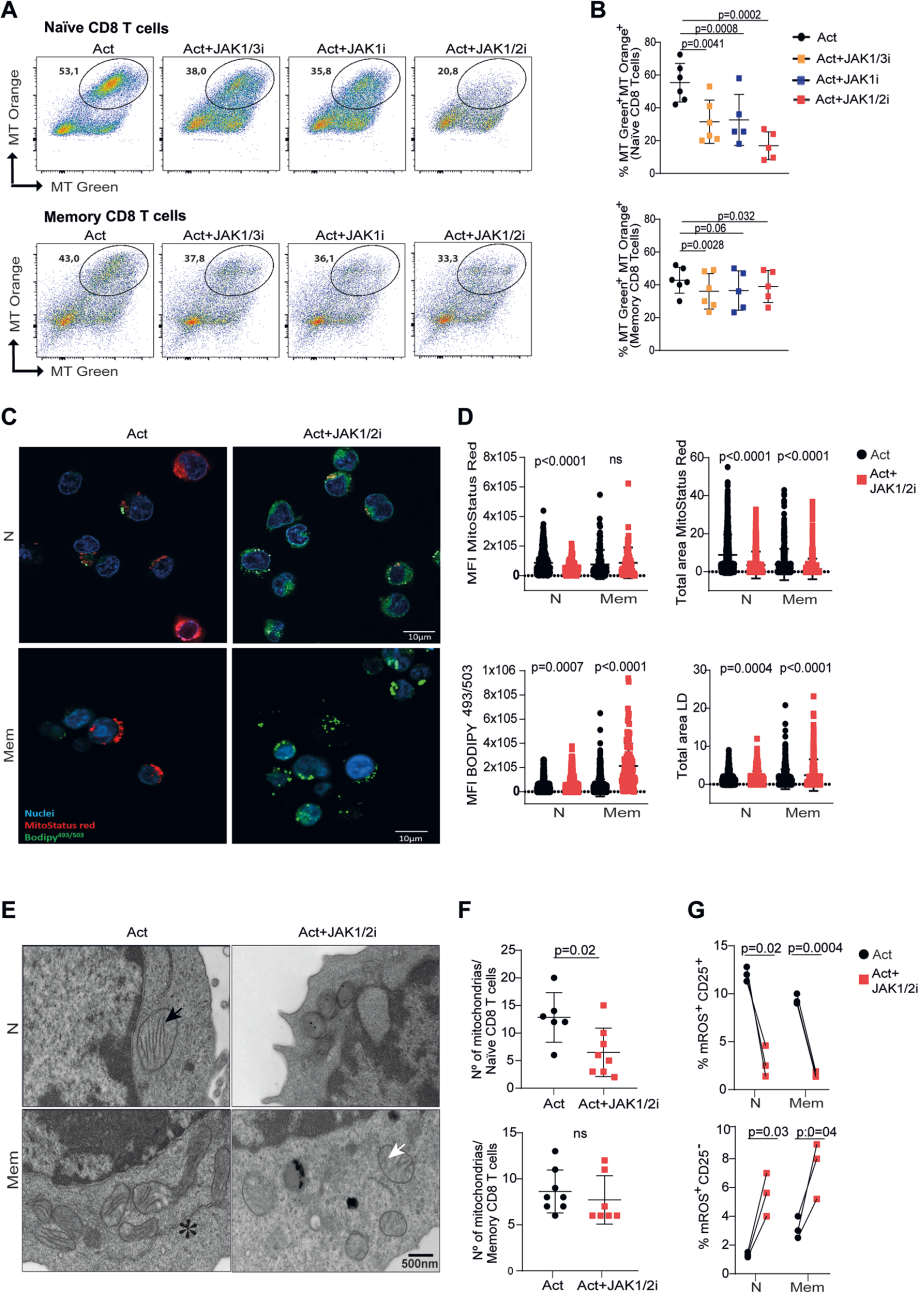
Mitochondrial polarisation, lipid accumulation, and ultrastructural alterations in CD8 T cells treated with JAK inhibitors. Naïve and memory CD8 T cells from healthy donors (HD) were stimulated for 3 days in the presence or absence of JAK1/3i, JAK1i, or JAK1/2i and evaluated using flow cytometry, confocal microscopy, and TEM. **A**, **B** Representative dot plots (**A**) and pooled data (**B**) of MT Green⁺ MT Orange⁺ cells; upper panel: naive CD8 T cells, lower panel: memory CD8 T cells. Polarised mitochondria were defined as MT Green⁺MT Orange⁺. **C**, **D** Confocal microscopy images of CD8 T cells stained with MitoStatus Red, a Δψm-dependent probe, and BODIPY493/503. **C** Quantification of mitochondrial parameters, including total area and mean fluorescence intensity of MitoStatus Red and lipid droplet content, including total area and mean fluorescence intensity of BODIPY, is presented in (**D**). Measurements were performed by high-content analysis, allowing quantitative comparison of mitochondrial polarisation and lipid accumulation across naive and memory CD8 T cells under each condition. **E** Transmission electron microscopy images (×15,000) of activated naive and memory CD8 T cells cultured with or without JAK1/2i. Black arrowheads: mitochondria with well-defined cristae; white arrowheads: mitochondria with disrupted cristae; asterisks: rough endoplasmic reticulum. **F** Quantification of mitochondrial number per cell from TEM images. **G** Frequency of CD25⁻mROS⁺ and CD25⁺mROS⁺ CD8 T cells under the indicated conditions. Data in (**B**) are presented as mean ± SD from ine individual HD and assayed across three independent experiments. Data in (**C**, **D**) are representative of two independent experiments with 2 HD. Statistical significance in (**B**) was assessed using mixed-effects analysis followed by Dunnett’s multiple comparisons test. Paired *t* test was used in (**G**); unpaired *t* test was applied in (**D**, **F**).

Under αCD3/αCD28 stimulation, a large fraction of naive and memory CD8 T cells displayed polarised mitochondria, defined as MitoTracker Orange⁺ MitoTracker Green⁺ by flow cytometry, indicating mitochondria that maintain intact Δψm. The presence of JAK1/3i, JAK1i, or JAK1/2i reduced this fraction, with a stronger effect in naive cells (Fig. 3A, B). Confocal microscopy using MitoStatus Red, a Δψm-dependent probe, corroborated these findings, showing a smaller total area of polarised mitochondria and lower mean fluorescence intensity in JAKi-treated naive and memory CD8 T cells (Fig. 3C, D). These results indicate reduced mitochondrial polarisation and lower Δψm under JAK inhibition during CD8 T-cell activation.

In addition to glucose metabolism, fatty acid β-oxidation serves as an alternative energy source in T cells, particularly when glycolysis is restricted [30]. To determine whether JAK inhibition perturbs lipid handling, we analysed neutral lipid content. Both naive and memory CD8 T cells activated in the presence of JAKis showed greater accumulation of lipid droplets, reflected by increased BODIPY^503/493^ MFI and area (Fig. 3C, D). These findings suggest that JAKis promote lipid storage or impair lipid utilisation during activation.

To further assess the impact of JAK inhibition on organelle remodelling, we performed transmission electron microscopy (TEM) of naive and memory CD8 T cells cultured with or without JAK1/2i, used here as a representative JAKi. In control, activated naive and memory CD8 T cells displayed abundant mitochondria with elongated shape and densely packed cristae, along with expanded cytoplasm and well-developed rough endoplasmic reticulum cisternae (Fig. 3E and Fig. S6A, left panels). In contrast, JAK1/2i-treated CD8 T cells showed reduced activation-associated ultrastructural features. Mitochondria were frequently swollen, with fragmented cristae and electron-lucent regions within the matrix, consistent with structural impairment (Fig. 3E and Fig. S6A, right panels). Quantitative analysis showed fewer mitochondria per naive CD8 T-cell in the presence of JAK1/2i (Fig. 3F).

During clonal expansion, mROS act as signalling intermediates that support T-cell proliferation when kept within an optimal range [31, 32]. Stratifying cells by CD25 upregulation, an AIM marker, revealed distinct effects of JAKis on mROS levels depending on activation status. In CD25⁺ cells, JAK1/2i markedly reduced mROS production, whereas CD25⁻ cells displayed increased mROS compared to their counterparts without inhibitors (Fig. 3G and Fig. S6B), highlighting that JAKis differentially modulate mitochondrial signalling depending on the activation state.

Together, these ultrastructural and functional data demonstrate that JAK inhibition compromises the mitochondrial and morphological remodelling required for CD8 T-cell activation, highlighting its impact across multiple layers of metabolic adaptation.

### JAK1/2i drives metabolic stress and senescence-like transcriptional programmes in memory CD8 T cells

Since the three JAKis impaired activation and effector function in both naive and memory CD8 T cells, we next sought to explore in depth the impact of a representative inhibitor at the transcriptional level. We selected JAK1/2i and focused on memory CD8 T cells, as this subset plays a central role in antiviral immunity and secondary responses. To this end, we performed RNA-seq on AIM⁺ memory CD8 T cells, sorted as shown in Fig. S7A, to characterise the transcriptional programmes affected by JAK1/2i in cells that effectively engaged activation. Differential expression analysis identified 965 genes with significant changes ( | Log₂FC | ≥ 2, *P* < 0.01) between JAK1/2i-treated and control conditions (Fig. S7B). To validate our system and analytical approach, we first confirmed that curated JAK-STAT–dependent gene sets such as IL-2 signalling and IFN-γ response were significantly downregulated by JAK1/2i (Fig. S7C, D). In addition, gene sets associated with cell cycle progression, including G0 to G1 transition and DNA strand elongation, were suppressed, consistent with a transcriptomic signature of cell cycle arrest.

**Fig. 4:**
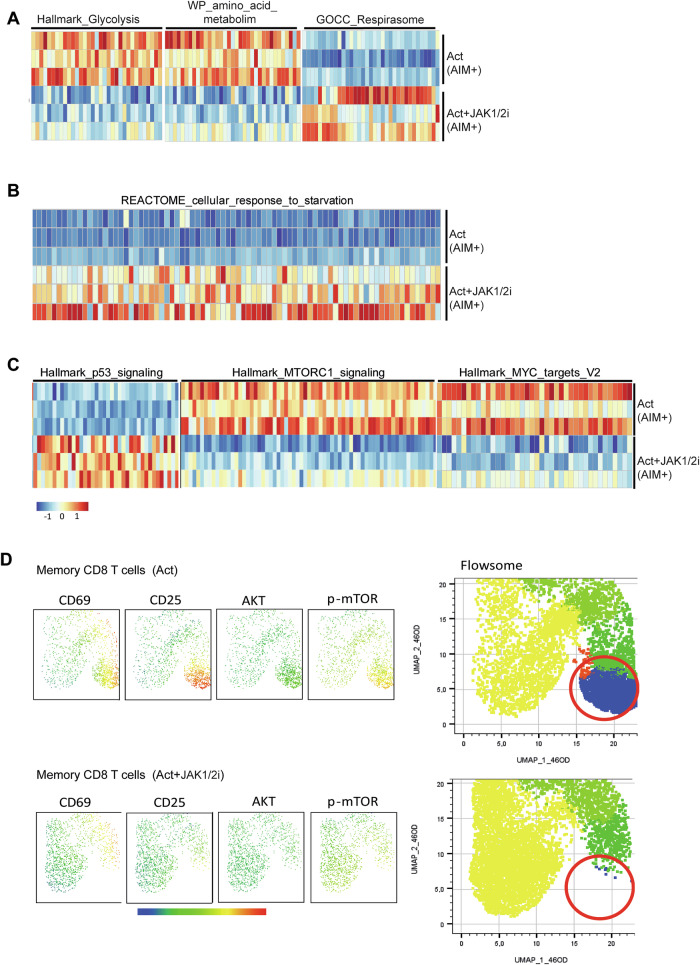
Transcriptomic and proteomic analyses of key metabolic pathways in memory CD8 T cells treated with JAK1/2i. **A**–**C** Heatmaps of leading-edge genes derived from Gene Set Enrichment Analysis (GSEA) of RNA-seq data from AIM⁺ memory CD8 T cells, which had been stimulated for 3 days with or without JAK1/2i prior to sorting. **A** Gene sets related to glycolysis, amino acid metabolism, and mitochondrial respiration. **B** Gene set associated with the cellular response to starvation. **C** Hallmark gene sets related to p53 signalling, mTORC1 signalling, and MYC targets. Data in (**A**–**C**) are from RNA-seq performed with three independent donors. **D** Representative UMAP and FlowSOM analysis of activation markers (CD69⁺CD25⁺) and p-AKT and p-mTOR in memory CD8 T cells. Data in (**A**–**C**) are from RNA-seq performed with three independent donors. Data in (**D**) correspond to flow cytometry analyses from three donors, shown as one representative experiment out of three performed.

Having established that canonical JAK-dependent pathways were effectively inhibited, we next interrogated metabolic programmes. GSEA revealed that JAK1/2i significantly downregulated gene sets associated with glycolysis and amino acid metabolism (Fig. 4A and Fig. S8A), indicating suppression of key anabolic processes. In contrast, the gene set related to mitochondrial respiration was upregulated (Fig. 4A and Fig. S8A), suggesting enhanced oxidative phosphorylation. Consistent with this, JAK1/2i-treated cells also showed enrichment of a starvation response signature (Fig. 4B and Fig. S8B), supporting the induction of a metabolic stress programme in activated memory CD8 T cells. These findings further connect JAK inhibition to senescence-like programmes in T cells.

In recent years, the crosstalk between the tumour suppressor protein p53 and mTOR pathways has been extensively described, with p53 acting as a negative regulator of cell growth by inhibiting mTOR and related pathways such as MYC under stress conditions [33]. In line with this, transcriptomic analysis revealed increased expression of hallmark p53 signalling genes together with a downregulation of mTORC1 and MYC target signatures in JAK1/2i-treated memory CD8 T cells (Fig. 4C and Fig. S8C). Importantly, these transcriptomic findings were supported by phospho-protein expression measured by flow cytometry in CD8 T cells. UMAP and FlowSOM analyses showed a marked reduction in p-AKT and p-mTOR expression across specific activated clusters of memory CD8 T cells (Fig. 4D). This was further validated by side-by-side comparisons of p-mTOR and p-AKT expression in naive and memory CD8 T cells (Fig. S9A, B), which showed a significant reduction with all three JAKis at pharmacokinetic concentrations. When restricting the analysis to AIM^+^ CD8 T cells, we observed consistent decreases in both p-mTOR and p-AKT across all JAKis, whereas AIM^-^ CD8 T cells displayed no detectable changes (Fig. S9C, D). Together, these results indicate that JAK1/2i enforces a transcriptional and proteomic programme of metabolic stress in memory CD8 T cells, linking metabolic rewiring to stress and growth-inhibitory signalling. This suggests a mechanism through which JAK inhibition imposes senescence-like constraints on activated memory CD8 T cells.

### JAK1/2i reduces protein synthesis and promotes mitochondrial dependence in activated memory CD8 T cells

Building on the transcriptomic and flow cytometry-based phospho-protein data pointing to reduced mTOR activity and altered metabolic programmes, we next asked whether these changes translated into functional consequences at the single-cell level. For this purpose, we used the SCENITH assay, a flow cytometry-based method that measures protein synthesis through puromycin incorporation and simultaneously infers cellular dependence on glycolysis or mitochondrial respiration [34].

**Fig. 5:**
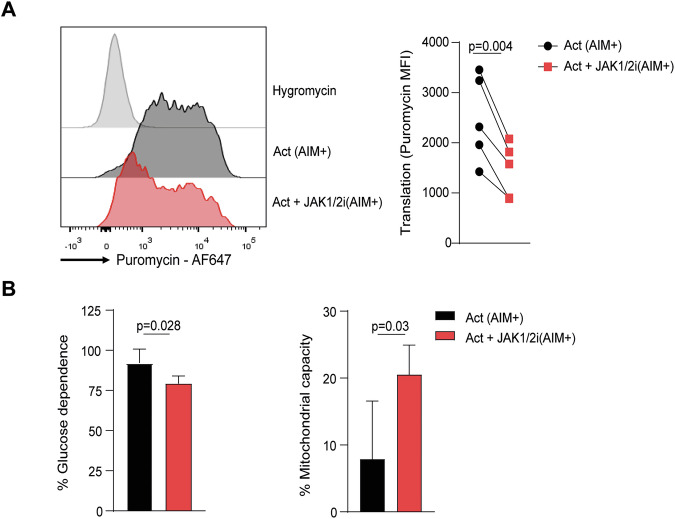
Functional impact of JAK1/2ion protein synthesis and metabolic dependence in activated memory CD8 T cells. SCENITH assay was performed on memory CD8 T cells stimulated for 3 days in the presence or absence of JAK1/2i. **A** Representative histograms of puromycin incorporation and hygromycin controls (left), together with pooled data of puromycin incorporation in AIM⁺ memory CD8 T cells (right). **B** Quantification of glucose dependence and mitochondrial capacity in AIM^+^ memory CD8 T cells under the indicated conditions. Data are presented as mean ± SD from five individual HD assayed across two independent experiments. *P* values were calculated using paired *t* test (**A**, **B**).

Upon activation, robust puromycin incorporation was observed in AIM^+^ memory CD8 T cells compared to the negative control with hygromycin (Fig. 5A). When treated with JAK1/2i, AIM⁺ cells showed a marked reduction in puromycin incorporation, indicating diminished global protein synthesis. In addition, JAK1/2i-treated AIM^+^ cells displayed lower glucose dependence and increased mitochondrial capacity (Fig. 5B), consistent with a shift in their bioenergetic profile. Together, these findings provide functional evidence that JAK1/2i reduces protein synthesis and reprogrammes energy metabolism in activated memory CD8 T cells.

### JAKi therapy in RA recapitulates defects in metabolism and protein translation in memory CD8 T cells

**Fig. 6:**
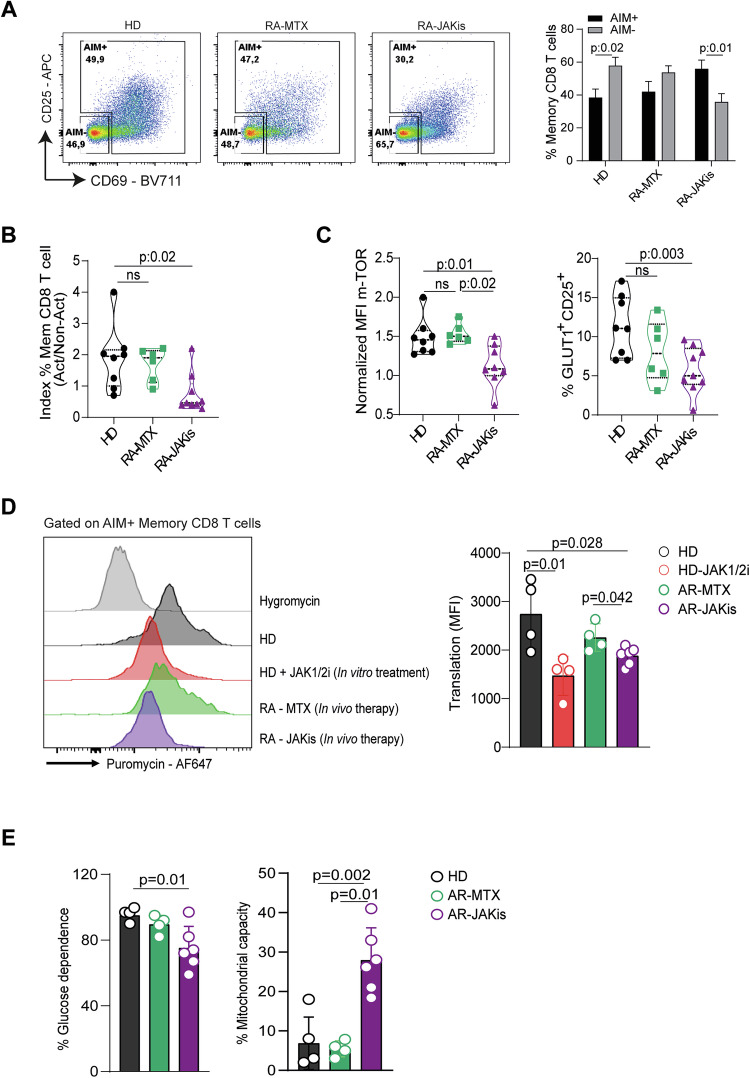
JAK inhibition in RA patients recapitulates metabolic and translational defects in memory CD8 T cells. PBMCs from healthy donors (HD) and RA patients treated with methotrexate (RA-MTX) or JAK inhibitors (RA-JAKis) were stimulated with PHA for 24 h. **A** Representative dot plots (left) and pooled frequencies (right) of AIM⁺ (CD25⁺ and/or CD69⁺) versus AIM⁻ memory CD8 T cells. **B** Ratio of AIM^+^ to AIM^-^ memory CD8 T cells in HD, RA-MTX, and RA-JAKis groups. **C** Normalised MFI of p-mTOR and frequency of GLUT1⁺CD25⁺ cells among memory CD8 T cells. **D** Puromycin incorporation in AIM^+^ memory CD8 T cells from HD, RA-MTX, and RA-JAKis patients; cells from HD stimulated in the presence of JAK1/2i were included for comparison. Left, representative histograms with hygromycin controls; right, pooled data. **E** Quantification of glucose dependence and mitochondrial capacity in AIM^+^ memory CD8 T cells. Data are presented as mean ± SD. One-way ANOVA followed by Tukey’s multiple comparisons test was used for comparisons in (**B**–**E**), except for the in vitro control comparison in (**D**) (HD vs HD+JAK1/2i), where a paired *t* test was applied.

To extend our in vitro observations to a clinically relevant context, we compared PBMCs from HD and RA patients treated with JAKis or methotrexate (MTX), a conventional DMARD with a distinct mechanism of action (Table 1). In this setting, PBMCs were stimulated with PHA for 24 h, a condition chosen to better preserve the ex vivo metabolic status of patient T cells. Flow cytometric analysis of CD69 and CD25 expression defined AIM⁺ and AIM⁻ memory CD8 T cells (Fig. 6A). HD and RA-MTX samples showed a relatively balanced distribution, whereas RA patients under JAKi therapy exhibited fewer AIM⁺ and more AIM⁻ cells, resulting in a lower AIM⁺/AIM⁻ ratio (Fig. 6A, B). Consistently, RA patients under JAKi therapy showed reduced p-mTOR expression on memory CD8 T cells and a smaller fraction of GLUT1⁺CD25⁺ cells within this subset (Fig. 6C), consistent with impaired metabolic activation.

**Table 1:** Demographic, biochemical and clinical characteristics of healthy donors and RA patients.

	HD	DMARDs-MTX	JAKis
*n*	9	8	10
Sex (F/M)	7/2	6/2	7/3
Age range (years)	30–58	33-68	30-77
ESR mm/h (median±QD)		4 ± 1.5	5.5 ± 8.5
CRP mg/L (median±QD)		1.1 ± 9	1.2 ± 2.5
DAS28 (mean ± SD)		2.4 ± 1	2.5 ± 1.1
RF (+/−)		8/0	10/0
Anti-CCPs (+/−)		5/3	9/1

*Anti-CCPs* anti-cyclic citrullinated proteins, *CRP* C-reactive protein, *DAS* disease activity score, *DMARDs* disease-modifying antirheumatic drugs, *ESR* erythrocyte sedimentation rate, *HD* healthy donors, *QD* quartile deviation, *RF* rheumatoid factor, *Tofa* tofacitinib, *uTx* untreated.

We then assessed translational capacity using SCENITH. In line with our in vitro findings, AIM^+^ memory CD8 T cells from RA patients treated with JAKis incorporated significantly less puromycin than those from HD or RA patients treated with MTX (Fig. 6D). Puromycin incorporation in memory CD8 T cells from RA patients on MTX therapy resembled that of HD T cells, whereas cells from RA patients on JAKis overlapped with HD T cells stimulated with PHA in the presence of JAK1/2i, which was included as an additional control. Finally, memory CD8 T cells from RA patients treated with JAKis also showed reduced glucose dependence and increased mitochondrial capacity (Fig. 6E), indicating a shift toward oxidative metabolism.

Altogether, these results demonstrate that the impaired p-mTOR activity, reduced glycolytic reliance, and defective protein synthesis observed in vitro are recapitulated in RA patients treated with JAKis, reinforcing the concept that memory CD8 T cells are direct targets of JAKi-mediated metabolic rewiring.

## Discussion

Although JAK inhibitors are widely used in the clinic, their impact on the metabolic machinery that fuels T-cell effector function is largely unknown. Because metabolism is a key determinant of cytotoxicity and cytokine production, understanding how these drugs reshape metabolic engagement is essential. Here, we show that JAK inhibition uncouples phenotypic activation from the metabolic programmes required for effector function, revealing disruption of key metabolic checkpoints that sustain differentiation. Importantly, the reduced expression of effector molecules observed under JAK inhibition was accompanied by a marked impairment in CD8 T-cell cytotoxic activity, highlighting a direct functional consequence of disrupted effector differentiation.

A well-established paradigm is that T-cell activation requires the coordinated engagement of glycolysis, mitochondrial respiration, and growth pathways such as mTOR and MYC [14, 17]. In contrast, we observed that both naive and memory CD8 T cells activated under JAKi treatment could upregulate activation markers but failed to mount a robust glycolytic response. This effect was observed not only with inhibitors targeting different combinations of JAK1, JAK2, and JAK3, but also upon TYK2 inhibition, suggesting a convergent impact of JAK family blockade on T-cell metabolic reprogramming. Notably, we observed a similar uncoupling between activation and glycolytic reprogramming in CD4 T cells, indicating that this effect is not restricted to the CD8 compartment. While the present study focuses on T cells, and particularly on CD8 T cells as a mechanistic model given their central role in antimicrobial immunity and host defence, these findings raise the possibility that JAK inhibition may affect metabolic engagement across additional immune cell populations, an aspect that warrants further investigation.

While memory CD8 T cells are normally metabolically poised to respond more rapidly than naive cells [35], our findings indicate that JAK inhibition blunts this readiness, limiting the energetic and biosynthetic support needed for effective proliferation and cytokine production. Within the framework of Signal 4, whereby nutrients provide a licensing signal in concert with TCR, costimulation, and cytokines, our findings suggest that JAK inhibition disrupts this nutrient-driven licensing. Specifically, impaired glycolysis together with repression of mTOR/MYC programmes indicates that CD8 T cells fail to receive the full metabolic “green light” required for effector differentiation [36]. This uncoupling between phenotype and metabolism raises the key question of whether JAK inhibition merely delays metabolic engagement or instead imprints a more permanent metabolic scar that constrains effector differentiation. In either case, impaired glycolysis is expected to compromise antiviral activity, given the tight link between glycolytic flux and cytotoxic T-cell functions [37], a notion supported by the reduced CD8 T-cell cytotoxic activity observed under JAK inhibition.

Reduced glycolytic activity under JAK inhibition may have consequences beyond impaired effector function. A body of evidence links glycolytic restriction to the induction of senescence. For example, epithelial cells undergoing oncogene-induced senescence show decreased glucose uptake and lactate production, and pharmacological inhibition of glycolysis accelerates senescence, whereas sustained HK2-dependent glycolysis allows escape from growth arrest [38, 39]. These findings support the concept that glycolytic insufficiency cannot only restrain effector differentiation but also initiate senescence-like programmes, providing a mechanistic bridge between metabolic stress and the phenotypes we observed under JAK inhibition.

Consistent with this broader picture of metabolic stress, we found that JAKis also impaired mitochondrial structure and function while driving a relative increase in OXPHOS dependence. Rather than contradictory, these findings suggest an adaptive response whereby cells with impaired mitochondrial capacity, yet with glycolysis strongly repressed, become more dependent on OXPHOS as a survival strategy. The observed reduction in mitochondrial membrane potential and fragmented cristae is consistent with structural vulnerability, whereas the increased reliance on oxidative metabolism indicates an attempt to preserve viability under conditions of restricted glycolysis. Such a state of adaptive stress could predispose CD8 T cells to metabolic senescence, limiting their longevity and functional versatility. Supporting this view, T cells harbouring genetically driven mitochondrial dysfunction have been shown to precipitate senescence and ageing-related features in mice [40].

Mechanistically, these alterations were associated with repression of mTOR/MYC-driven programmes together with induction of p53, consistent with a transition from anabolic growth toward metabolic stress and senescence pathways [33]. This shift is in line with our previous work showing that JAKis trigger DNA damage responses in memory T cells [20]. Overall, our results highlight that senescence-like constraints are a central outcome of JAK inhibition, linking metabolic rewiring to impaired T-cell fate decisions. Whether the senescence-like phenotype we observed is reversible or represents a fixed fate remains an open question with major implications for secondary responses. If persistent, this phenotype could compromise the ability of patients on JAKis to generate effective recall responses, providing a mechanistic explanation for their increased susceptibility to infections. Importantly, our in vitro findings converged with data from RA patients receiving JAKis, who displayed reduced p-mTOR, impaired protein synthesis, and a metabolic bias toward OXPHOS. This consistency underscores the translational relevance of our observations and, although based on a limited cohort, shows that the effects were reproducible and biologically meaningful. Building on this proof of concept, our findings suggest that metabolic profiling approaches such as SCENITH, or surrogate markers of T-cell metabolic activation, could be explored as biomarkers to stratify patients at higher risk of infection during JAKi therapy.

Finally, our results broaden the discussion beyond antiviral immunity. We observed lipid droplet accumulation in memory CD8 T cells activated under JAK inhibition, consistent with altered lipid handling. Lipid droplets are increasingly recognised as hubs of immune and metabolic regulation, and their dysregulation has been linked to inflammatory and cardiovascular pathologies [41]. Although these changes may represent an adaptive response, impaired lipid utilisation could also affect the fitness and function of other immune cell lineages, raising the possibility of broader metabolic consequences under JAKi therapy. While these considerations remain speculative, they are consistent with clinical observations reporting thromboembolic and cardiovascular events in JAKi-treated patients [42, 43], and warrant further investigation across cell types and clinical contexts.

Together, these findings indicate that metabolic rewiring is a central mechanism of JAK inhibition. This provides a unifying framework that links impaired immune fitness with senescence-like programmes, with implications for both host defence and systemic health. Our study has certain limitations, including the relatively small patient cohort, the primary focus on CD8 T cells, and the absence of antigen-specific analyses or correlation with adverse clinical outcomes. Nonetheless, the convergence of experimental and patient data offers a clear proof of concept and provides a strong rationale for future studies aimed at expanding patient cohorts, defining biomarkers of risk, and clarifying the clinical impact of metabolic rewiring under JAKi therapy.

## Materials and methods

### Study subjects and samples

Healthy donors (HD) were enrolled as volunteers at the Facultad de Ciencias Químicas, Universidad Nacional de Córdoba (Argentina). Exclusion criteria included any history of autoimmune disease or use of immunosuppressive therapy.

RA patients receiving methotrexate or JAKi therapy were recruited from the Rheumatology Service (Instituto Médico Strusberg, Córdoba, Argentina). Diagnosis was established according to the American College of Rheumatology (ACR) and the European League Against Rheumatism (EULAR) criteria [44]. Exclusion criteria included ongoing infections or metabolic diseases. Demographic and clinical characteristics of all study participants are summarised in Table 1. Clinical laboratory parameters (ESR, CRP, RF, anti-CCP) were determined using standard procedures as previously described [20].

Peripheral blood (PB) samples were collected from HD and RA patients, and peripheral blood mononuclear cells (PBMCs) were isolated by density-gradient centrifugation using Ficoll- Hypaque (GE Healthcare/Cytiva-Ficoll-Paque™ PLUS).

### Cell sorting

Fresh PBMCs were stained with anti-CD8 (PerCP, UCHT-4, ImmunoTools, Cat: 21620085; or Alexa Fluor 700, RPA-T8, eBioscience, Cat: 56-0088-42), anti-CD45RA (FITC, ALB11, Beckman Coulter, Cat: A07786; or PE-Cy7, 5H9, BD, Cat: 561216), and anti-CCR7 (PerCP-Cy5.5, G043H7, BioLegend, Cat: 353220; or PE-Cy7, 3D12, BD, Cat: 557648). Different fluorochrome combinations were used depending on subsequent measurements to minimise spectral overlap. Naive (CCR7⁺CD45RA⁺) and memory (CCR7⁺CD45RA^−^/CCR7⁻CD45RA⁺/CCR7⁻CD45RA⁻) CD8 T cells were purified on a BD FACSAria II. Post-sort purity was assessed by flow cytometry and was typically >95%. The gating strategy for naive and memory CD8 T-cell subsets is shown in Fig. S1A.

### In vitro stimulation

Sorted naive and memory CD8 T cells (0.2 × 10^6^) were stimulated in Costar 96-well flat-bottom plates (Corning) precoated overnight with 1 μg/mL anti-CD3 (OKT3, eBioscience, Cat: 16-0037-85) and 0.5 μg/mL anti-CD28 (CD28.2, eBioscience, Cat: 16-0289-85) in the presence or absence of JAK inhibitors at concentrations corresponding to reported peak plasma levels ~2 h after oral administration in patients. Specifically, cells were treated with 0.2 μM tofacitinib [45] (Selleckchem) a JAK1/3i, 0.37 μM upadacitinib [23] (Selleckchem), a selective JAK1i, or 0.15 μM baricitinib [24] (Selleckchem), a JAK1/2i, or the TYK2 inhibitor deucravacitinib [46] (0.2 or 1 μM). Deucravacitinib was obtained as a commercially available 6 mg tablet, and processed following the same procedure previously used for tablet-derived tofacitinib [20]: mechanically disaggregated using a mortar and pestle, dissolved in DMSO, and centrifuged for 15 min at 10,000 rpm; the resulting supernatant was used as a stock solution with an estimated concentration of 10 mM. In selected experiments, sorted naive and memory CD4 T cells were stimulated under identical conditions in the presence or absence of the JAK1/2 inhibitor baricitinib. Cells were cultured in RPMI complete medium (GIBCO) supplemented with 10% FBS (GIBCO), 1% L-Glutamine (GlutaMax, Thermo Fisher Scientific) and 100 mg/ml streptomycin and 100 U/ml penicillin (GIBCO). After 3 days, surface molecules, intracellular cytokines, intranuclear transcription factors, the proliferation marker Ki-67 and metabolic assays, such as the 2-NBDG glucose analogue uptake, were evaluated as described below.

### Evaluation of cytokine-producing and proliferating T cells

Naïve or memory CD8 T cells were stimulated with 50 ng/ml phorbol 12-myristate 13-acetate (PMA, Sigma-Aldrich) and 1 μg/ml ionomycin (Sigma-Aldrich) for 2 h in the presence of brefeldin A (GolgiPlug, BD). After culture, cells were washed and stained for 30 min at 4 °C with anti-CD25 SB780 (BC96, eBioscience, Cat: 78-0259-42) and anti-CD137 PE (4B4-1, eBioscience, Cat: 12-1379-42). Subsequently, cells were washed, fixed and permeabilised for 20 min at 4 °C using Cytofix/Cytoperm (BD). Cells were washed twice with Perm/Wash (BD) and stained for 40 min at room temperature with anti-TNF-BV605 (MAb11, Biolegend, Cat: 502936), anti-granzyme B (BV421, GB11, BD, Cat: 563389; or AF647, GB11, Biolegend, Cat: 515406). Similarly, proliferation was determined by expression of the intracellular proliferation marker Ki-67 (anti-Ki-67 eFluor660, SolA15, eBioscience, Cat: 17-5698-82; or AF488, B56, BD, Cat: 561165). Fixable Viability Stain 510 (BD) was used for the exclusion of dead cells. Data were acquired on a BD LSRFortessa Cell Analyzer. The analysis and illustrations were performed using FlowJo (version 10).

### CD8 T-cell cytotoxicity assay

The cytotoxic activity of human memory CD8 T cells was evaluated using a CD3-redirected cytotoxicity assay [25]. CD8 memory T cells were isolated from PBMC of HD and activated for 3 days with anti-CD3/CD28 stimulation in the presence or absence of JAK inhibitors. Following activation, CD8 effector T cells were washed to remove residual inhibitors. Target cells were labelled with Cell Proliferator eFluor™ 670 (eBioscience, Cat: 65-0840-85) and pre-incubated with anti-CD3 antibody (OKT3). Activated CD8 effector memory T cells were then co-cultured with target cells for 12 h at 37 °C at different effector-to-target (E:T) ratio. Target cell viability was assessed by flow cytometry using viability staining within the Cell Proliferator eFluor™ 670⁺ gate.

### Metabolic in vitro assays using flow cytometry

#### Glucose analogue uptake assay using 2-NBDG

Cells were plated in pre-warmed low glucose DMEM medium (Thermo Fisher Scientific), supplemented with 2% SBF and incubated for 30 min at 37 °C, 5% CO₂ atmosphere to consume residual glucose. Then, cells were incubated with 2-NBDG (Life Technologies, Cat. N13195) at a final concentration of 15 µM for 45 min at 37 °C, 5% CO₂ atmosphere in the dark. After incubation, cells were washed with PBS and stained for surface markers. Fluorescence was measured by flow cytometry (FITC channel).

#### MitoTracker staining

The assays were performed according to the manufacturer’s instructions (MitoTracker® Mitochondrion-Selective Probes). Briefly, cells were incubated with 100 nM MitoTracker Green (M-7514, Thermo Fisher Scientific) and 50 nM MitoTracker Orange CMTMRos (M7510, Thermo Fisher Scientific) in RPMI for 30 min at 37 °C for mitochondrial membrane potential and mass, respectively. Fixable Viability Stain 510 (BD) was used for the exclusion of dead cells.

#### Mitochondrial reactive oxygen species (ROS)

ROS were detected using MitoSOX™ Green (M36005, Thermo Fisher Scientific) [47]. The final concentration of the probe was 0.5 µM. Cells were incubated with the dye at 37 °C in the dark for 20 min. After incubation, cells were washed thoroughly with pre-warmed (37 °C) clean PBS to remove excess dye. Fixable Viability Stain 510 (BD) was used for the exclusion of dead cells.

#### SCENITH assay

SCENITH experiments were performed as previously described [34] using the SCENITH kit containing all reagents and anti-puromycin antibodies (https://www.scenith.com/). Briefly, naive or memory CD8 T cells from healthy donors (HD) were sorted and cultured as described in the In vitro cultures section. In parallel, short-term (24 h) cultures of PBMCs from HD or RA patients were stimulated for 24 h with phytohaemagglutinin (PHA). Cells were then treated for 30 min at 37°C in the presence of the indicated inhibitors of various metabolic pathways and puromycin. After incubation, puromycin was detected using a fluorescently labelled anti-puromycin monoclonal antibody (R4743L-E8) conjugated to AF647, and analysed by flow cytometry. For metabolic analysis of sorted naive or memory CD8 T cells, we stained with anti-CD25-SB780 (BC96, eBioscience, Cat: 78-0259-42) and anti-CD69–BV711 (FN50, BioLegend, Cat: 310944). For metabolic analysis of PBMCs, we used the following panel: anti-CD3-AF700 (UCHT1, BD, Cat: 557943); anti-CD8-APC-Cy7 (HIT8a, BioLegend, Cat: 300926); anti-CD45RA-PE (ALB11, Beckman Coulter, Cat: IM1834I); anti-CCR7-PE-Cy7 (3D12, BD, Cat: 557648); anti-CD25-SB780 BC96, eBioscience, Cat: 78-0259-42) and anti-CD69–BV711 (FN50, BioLegend, Cat: 310944). Fixable Viability Stain 510 (BD) was used to exclude dead cells. The impact of the various metabolic inhibitors was quantified as described [34].

### Glucose and lactate measurement

Glucose (g/L) and lactate (mmol/L) concentrations were determined in culture supernatants by enzymatic colourimetric assays (Wiener Lab, Rosario, Argentina), following the manufacturer’s instructions.

### Confocal and high-content microscopy

To evaluate mitochondrial membrane potential and lipid droplet accumulation, both naive and memory CD8 T cells (after 3 days of stimulation in culture, as described above) were washed and stained with MitoStatus Red (APC channel, BD Biosciences) at a final concentration of 100 nM in RPMI medium. Cells were incubated at 37 °C for 30 min in a total volume of 100 µL per well. After incubation, cells were washed with pre-warmed PBS (Gibco) and subsequently stained with BODIPY™ 493/503 (Thermo Fisher Scientific) at a final concentration of 1 µg/mL in RPMI medium, together with Hoechst 33342 (Thermo Fisher Scientific) at 10 µg/mL, for 15 min at 37 °C. Cells were then washed twice with RPMI and finally resuspended in RPMI.

Microscopy images were acquired at ×60 magnification using an InCell Analyzer 2500HS (GE Healthcare) equipped with a Nikon ×60/0.70 Plan Apo CFI/60 objective. Ten fields per condition from two HD were captured. Image analysis was performed using InCarta version 1.10 (GE Healthcare). In parallel, high-resolution images were acquired using a ZEISS LSM 980 confocal microscope from two independent experiments, and collapsed for visualisation using FIJI software (version 1.53k).

### Transmission electron microscopy (TEM)

TEM analysis was performed at the Electron Microscopy Facility of INICSA-CONICET, Facultad de Ciencias Médicas, Universidad Nacional de Córdoba. Cells cultured in vitro for three days as described above were then fixed with 2.5% glutaraldehyde/2% paraformaldehyde (EMS, Delta-Microscopies), post-fixed, dehydrated, and embedded in resin. Ultrathin sections were prepared using an ultramicrotome (Leica, Germany) with a diamond knife. Micrographs were acquired with a Hitachi HT 7800 transmission electron microscope operated at 80 kV. Quantitative analyses, including the number of mitochondria per cell, were performed using ImageJ software.

### RNA sequencing and analysis methodology

Sorted memory CD8 T cells from three HD were cultured and stimulated in the presence or absence of JAK1/2i for 3 days as previously described. On day 3, cells were stained with anti-CD69-PEeF610 (FN50, eBioscience, Cat: 61-0699-42) and anti-CD25-APC (BC96, BioLegend, Cat: 302610), and AIM⁺ (CD69⁺CD25⁺/CD69⁻CD25⁺/CD69⁺CD25^−^) and AIM⁻ (CD69⁻CD25⁻) populations were sorted as shown in Fig. S4. RNA extraction and sequencing (RNA-seq) were performed by GENEWIZ Multiomics & Synthesis Solutions (Azenta Life Sciences, NJ, USA).

Upon receipt of frozen cell pellets, RNAlater® was removed according to the manufacturer’s instructions. Total RNA was extracted using the RNeasy Plus Micro Kit (Qiagen) and quantified with Qubit (Thermo Fisher Scientific). cDNA synthesis and amplification were carried out using the SMART-Seq v4 Ultra Low Input Kit (Clontech), followed by library preparation with Illumina Nextera XT (transposase fragmentation and indexed PCR). Libraries were validated on an Agilent TapeStation, quantified by Qubit and qPCR (KAPA Biosystems), pooled, and sequenced on an Illumina NovaSeq (2×150 bp paired-end). FASTQ files were generated using bcl2fastq v2.20 allowing one index mismatch.

Quality control and trimming were performed with Fastp v0.23.4 [48]; reads with Phred >30 were retained and aligned to the human genome (ENSEMBL GRCh38.112) using SubRead v2.0.6 [49]. Gene-level counts were obtained with featureCounts v2.0.6 [50]. Differential expression analysis was performed with edgeR v4.2.2 [51] after filtering low-expression genes (counts >10 in ≥70% of samples, and *filterByExpr*). Data were normalised by the trimmed mean of M-values (TMM) method. A generalised negative binomial linear model was fitted, and differential expression was assessed using the quasi-likelihood F-test with empirical Bayes dispersion estimates. Differentially expressed genes (DEGs) were defined as |log₂FC | ≥ 2 and *P* < 0.01. GO enrichment for biological process (BP), cellular component (CC), and molecular function (MF) terms, as well as Gene Set Enrichment Analysis (GSEA) [52], were conducted on the normalised count matrix.

### Statistical analyses

Statistical analyses were performed with GraphPad Prism version 10 (GraphPad Software). *P* values < 0.05 were considered significant. The D’Agostino–Pearson omnibus normality test was initially performed to determine the distribution of the datasets. The statistical tests used are indicated in the figure legends.

## Supplementary information

Supplementary material- Clean version

Fig. S1

Fig. S2

Fig. S3

Fig. S4

Fig. S5

Fig. S6

Fig. S7

Fig. S8

Fig. S9

aj-checklist-CDDIS-25-6287R

## Data Availability

The data that support the findings of this study are available from the corresponding author upon reasonable request. The raw data generated in this study can be accessed through the NCBI Short Read Archive (SRA) using the accession ID PRJNA1336864 and the direct link http://www.ncbi.nlm.nih.gov/bioproject/1336864. The scripts and the data files used for analysis can be found in the following repository: https://github.com/daniloceschin/JAKis-metabolic-licensing-CD8.

## AI-assisted language editing

ChatGPT (OpenAI) was used exclusively to improve the clarity and grammar of the manuscript. The tool did not contribute to data analysis, interpretation, or scientific conclusions. All scientific content, methodology, and interpretations are the sole responsibility of the authors, and all AI-assisted edits were reviewed and approved by the authors before submission.
