# Supplementary material for: Disruption of metabolic licensing by JAK inhibitors constrains CD8 T cell activation and effector function
Source: Cell Death Dis. 2026 Mar 24;17(1):355. doi: 10.1038/s41419-026-08610-7 (PMC13039990; doi:10.1038/s41419-026-08610-7)
Supplement: Supplementary file 1 — Supplementary material- Clean version [file 41419_2026_8610_MOESM1_ESM.docx]

**Supplementary information**

**Figure S1. Impact of JAK inhibitors on CD8 T cell activation, proliferation, and effector molecules expression.** Naïve and memory CD8 T cells from healthy donors were polyclonally stimulated for 3 days (Act) in the presence or absence of JAK1/3i, JAK1i, JAK1/2i or TYK2 inhibitors. (A) Gating strategy used to identify naïve and memory CD8 T cell subsets based on CD45RA and CCR7 expression after selection of live, single CD8 T cells. (B) Representative histograms showing the Ki-67, CD25, CD137, TNF, and GZMB expression in naïve and memory CD8 T cells under control conditions and in the presence of each JAK inhibitor (1µM). (C) Representative histograms illustrating the dose–response effects of JAK inhibitors (0.1–3 µM) on the same markers after stimulation as in (A). (D) Representative histograms showing the expression of CD25, CD137, and Ki-67 in naïve and memory CD8 T cells stimulated in the absence or presence of the TYK2i at the indicated concentrations. Data are representative of 2 experiments.

**Figure S2. JAK inhibition impairs the cytotoxic activity of memory CD8 T cells.**
Memory CD8 T cells from healthy donors were polyclonally activated (Act) for 3 days in the absence or presence of JAK1/3i or JAK1/2i and then co-cultured with target cells at the indicated effector-to-target (E:T) ratios for 12 h. **(A)** Representative flow cytometry plots showing target cell death at different E:T ratios. **(B)** Quantification of normalized target cell viability. P values were calculated with One-way Anova, Tukey's multiple comparisons test. Data are representative of two independent experiments.

**Figure S3. Effect of JAK inhibitors on glucose uptake and GLUT1 expression in CD8 T cells.** Naïve (N) and memory (Mem) CD8 T cells from healthy donors were polyclonally stimulated for 3 days (Act) in the presence or absence of JAK inhibitors. (A) Percentage of 2-NBDG⁺ cells within N and Mem CD8 T cells in the absence or presence of JAK1/3i, JAK1i, or JAK1/2i. (B) Percentage of GLUT1⁺ cells within N and MemCD8 T cells under the same condition than in A. (C) Frequency of 2-NBDG⁺ and GLUT1⁺ cells within N and Mem CD8 T cells following activation in the absence or presence of TYK2i. **(D)** Glucose and lactate concentrations measured in culture supernatants from cell cultures in C. Data are shown as individual values with mean ± SD and are representative of 3 independent experiments. P values were calculated using paired t-test (A–D).

**Figure S4. JAK inhibition modulates glucose uptake and metabolism in naïve and memory CD4 T cells.** Naïve (N) and memory (Mem) CD4 T cells from healthy donors were polyclonally activated for 3 days (Act) in the absence or presence of JAK1/2i. **(A)** Representative flow cytometry plots showing AIM⁺ N and Mem CD4 T cells. (B) Frequency of CD4 T cells incorporating the glucose analog 2-NBDG or expressing GLUT1 within the population expressing at least one activation marker (AIM⁺). (C) Glucose and lactate concentrations measured in culture supernatants from the same conditions. Data are representative of two independent experiments.

**Figure S5. Effect of JAK1/2i on activation and metabolic markers** **in CD8 T cells.** Flow cytometry–based FlowSOM maps showing expression of CD69, CD25, IRF4, GLUT1, and HIF-1α in naïve (A) and memory (B) CD8 T cells (B) CD8 T cells from healthy donors (HD) after 3 days of polyclonal stimulation (Act) in the presence or absence ofJAK1/2i. Data are representative of 2 independent experiments.

**Figure S6. Effect of JAK inhibitors on mitochondrial ROS and ultrastructure in CD8 T cells.** (A) Transmission electron microscopy (TEM) images showing cellular ultrastructure of CD8 T cells after activation with or without JAK1/2i. (B) Frequencies of naïve and memory mROS-positive CD8 T cells within CD25⁺ and CD25⁻ subsets after 3 days of polyclonally activation ± JAK inhibitors Data are representative of 3 independent experiments.

**Figure S7.** **RNA-seq analysis of AIM^+^ CD8 T cells.** (A) Sorting strategy of AIM^+^ CD8 T cells for RNA-seq after 3 days of activation ±JAK1/2i. (B) Volcano plot displaying significantly differentially expressed genes between Act+JAK1/2i and Act AIM^+^ CD8 T cells, with upregulated (violet) and downregulated (turquoise) genes. (C, D) enrichment plots and heat maps of leading-edge genes from representative enriched pathways confirm drug activity, highlighting IL2–STAT5 signaling, interferon-γ response, cell cycle, and DNA replication pathways. Data from n = 3 donors, 1 experiment.

**Figure S8. GSEA enrichment plots of transcriptomic changes in AIM^+^ CD8 T cells exposed to JAK1/2i.** Representative GSEA enrichment plots of metabolism-related pathways in AIM^+^ CD8 T cells after 3 days of activation ± JAK1/2i. Normalized enrichment scores (NES) and FDR q-values are indicated for each pathway. Data from n = 3 donors, 1 experiment.

**Figure S9. Effect of JAK inhibitors on p-mTOR and p-AKT in AIM^+^ CD8 T cells.** (A, B) Representative histograms of p-mTOR and p-AKT in AIM^+^ versus AIM- CD8 T cells. (C, D) Percentages of naïve and memory p-mTOR⁺ or p-AKT⁺ AIM^+^ CD8 T cells after 3 days of activation ± JAK inhibitors. Data are representative of 3 experiments. P values were calculated using paired t-test (C,D).
