# Supplementary figures and images for: Disruption of metabolic licensing by JAK inhibitors constrains CD8 T cell activation and effector function
Source: Cell Death Dis. 2026 Mar 24;17(1):355. doi: 10.1038/s41419-026-08610-7 (PMC13039990; doi:10.1038/s41419-026-08610-7)

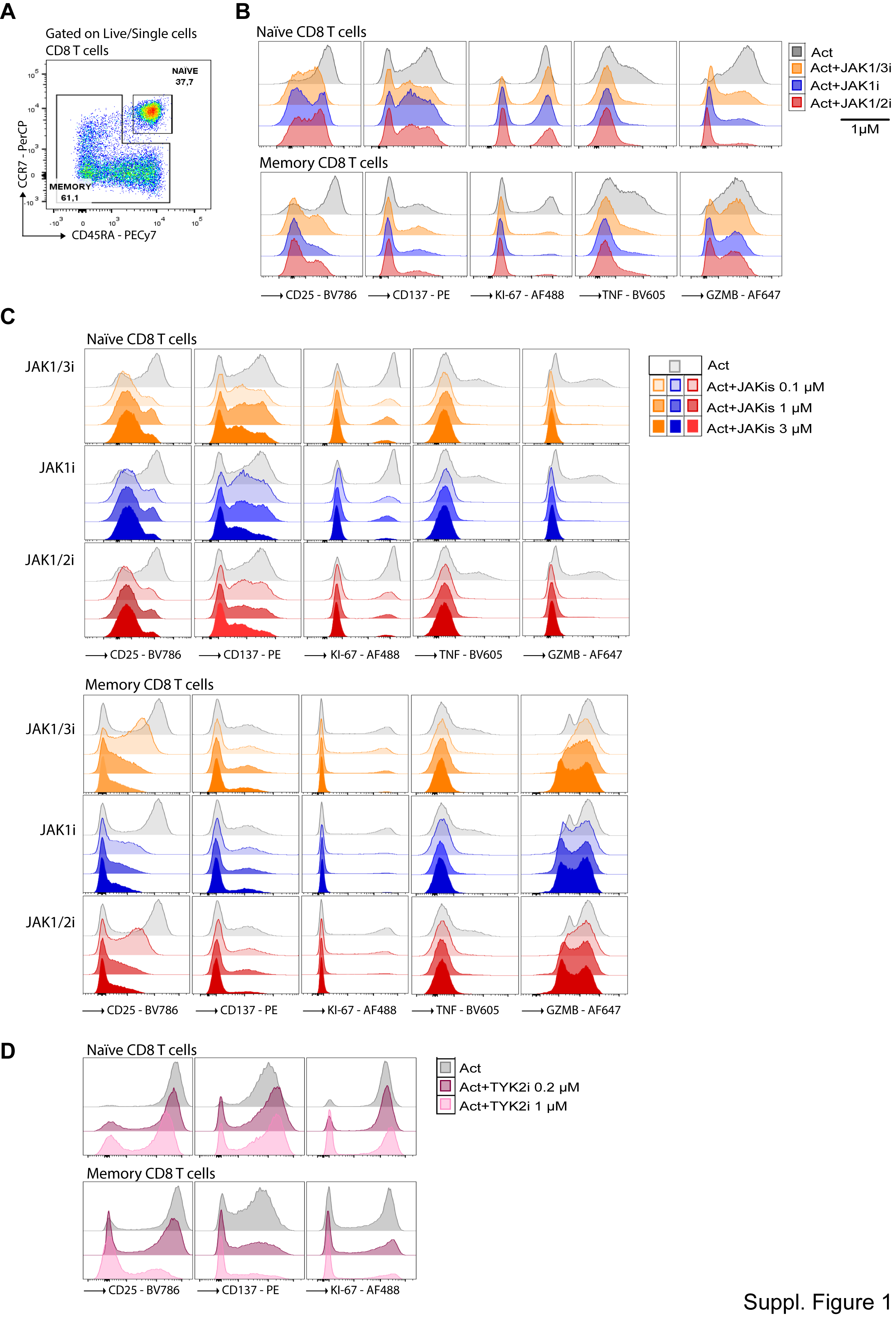

Supplement: Supplementary file 2 — Fig. S1 [file 41419_2026_8610_MOESM2_ESM.tif]

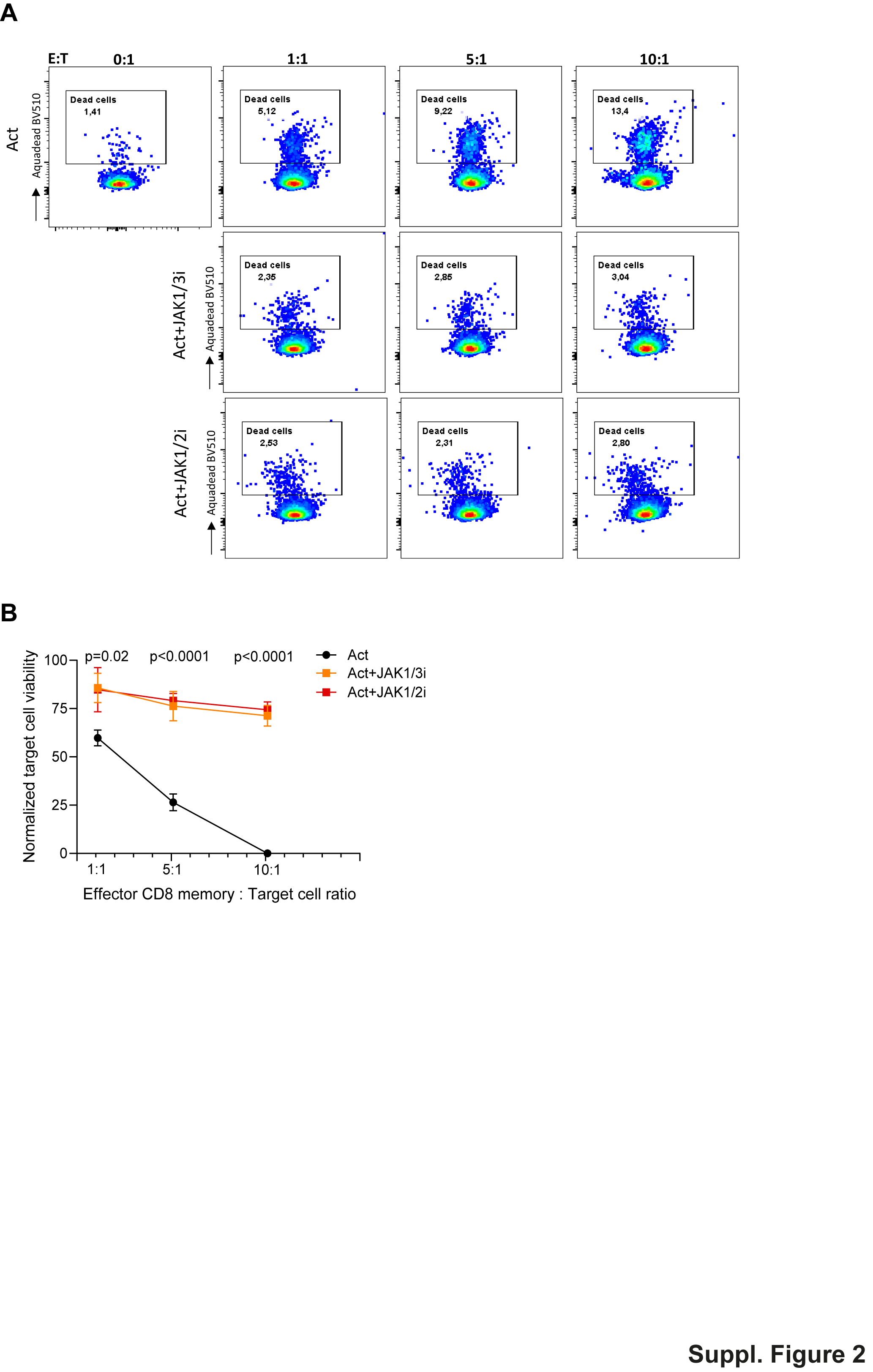

Supplement: Supplementary file 3 — Fig. S2 [file 41419_2026_8610_MOESM3_ESM.tif]

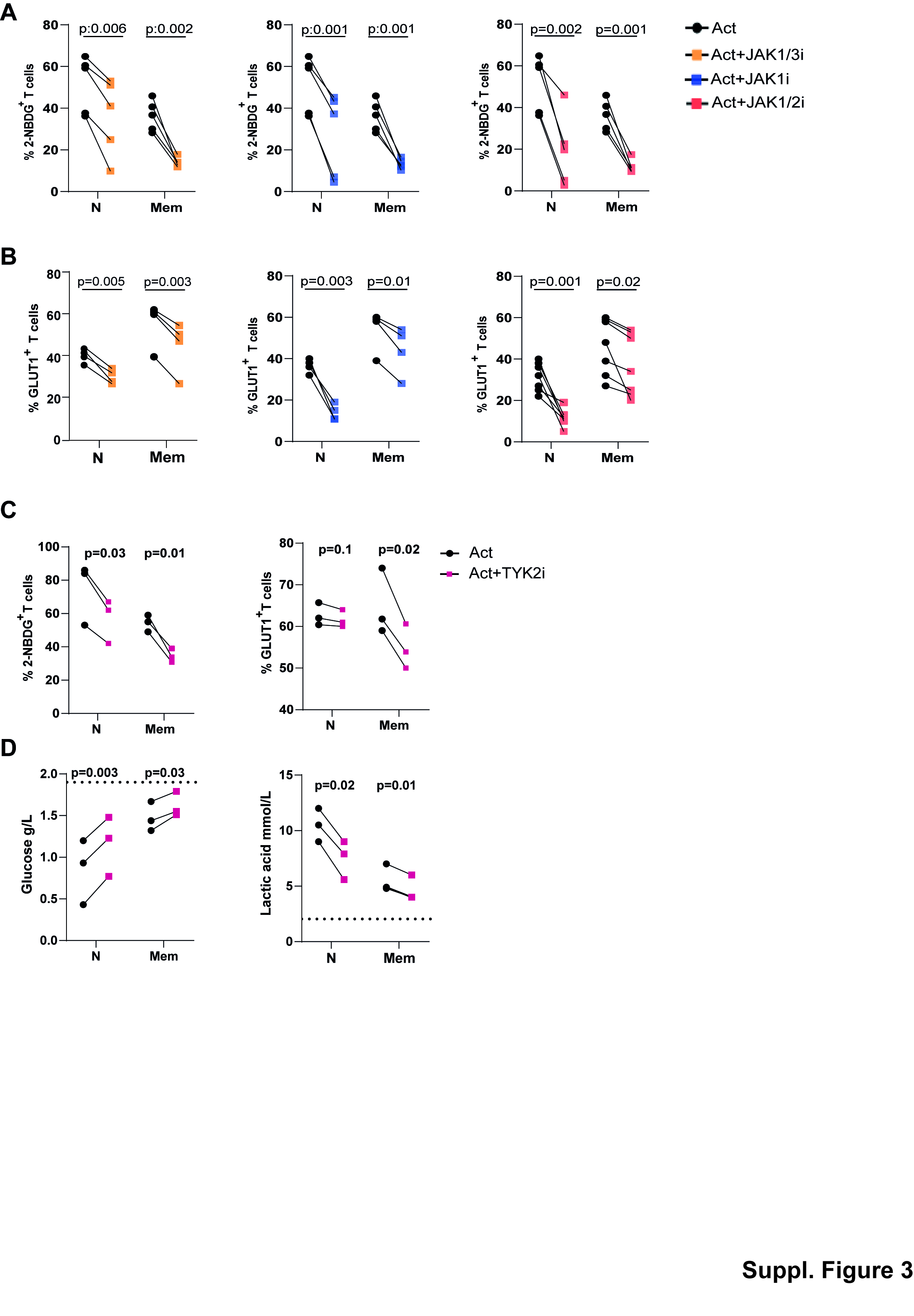

Supplement: Supplementary file 4 — Fig. S3 [file 41419_2026_8610_MOESM4_ESM.tif]

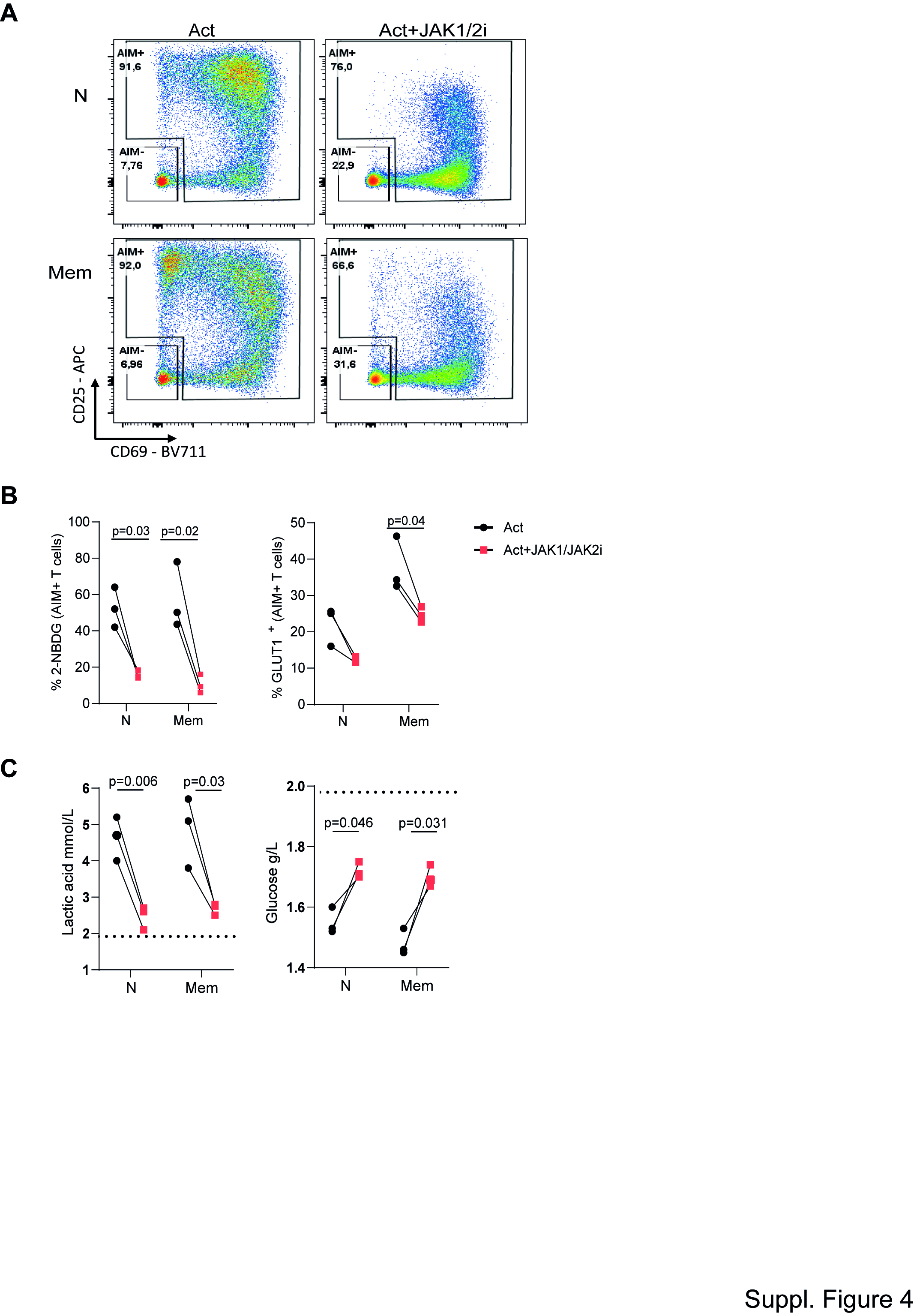

Supplement: Supplementary file 5 — Fig. S4 [file 41419_2026_8610_MOESM5_ESM.tif]

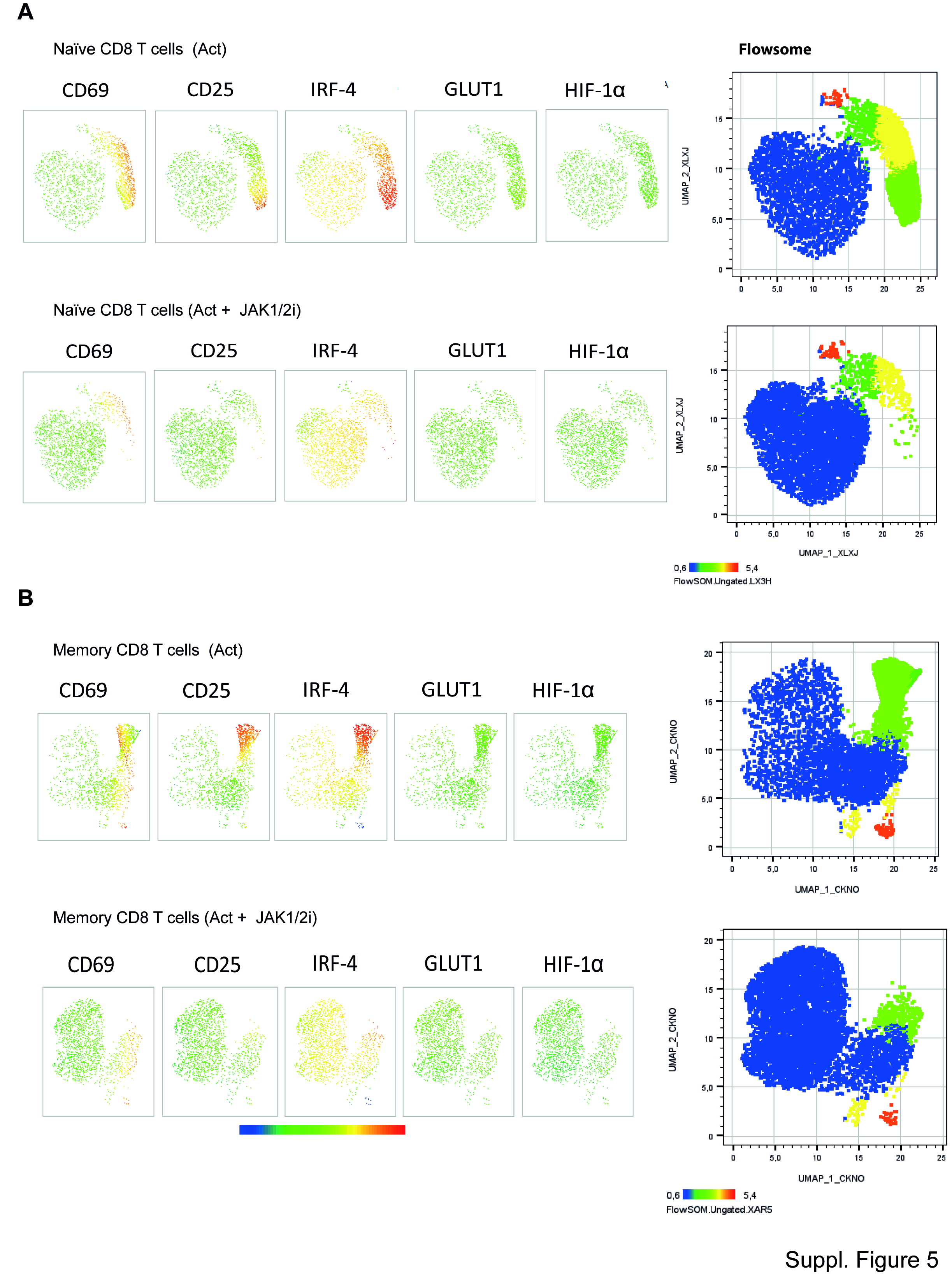

Supplement: Supplementary file 6 — Fig. S5 [file 41419_2026_8610_MOESM6_ESM.tif]

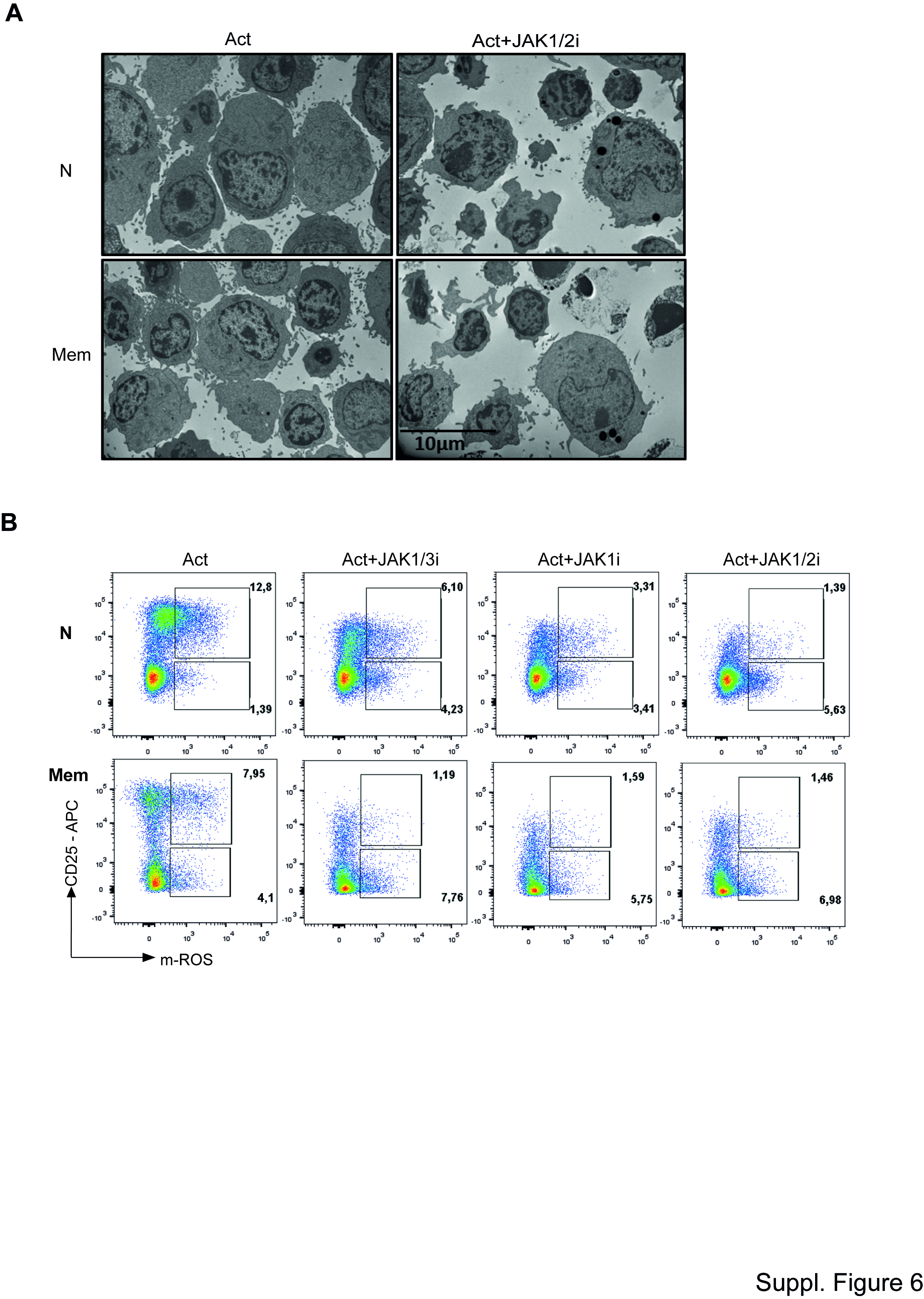

Supplement: Supplementary file 7 — Fig. S6 [file 41419_2026_8610_MOESM7_ESM.tif]

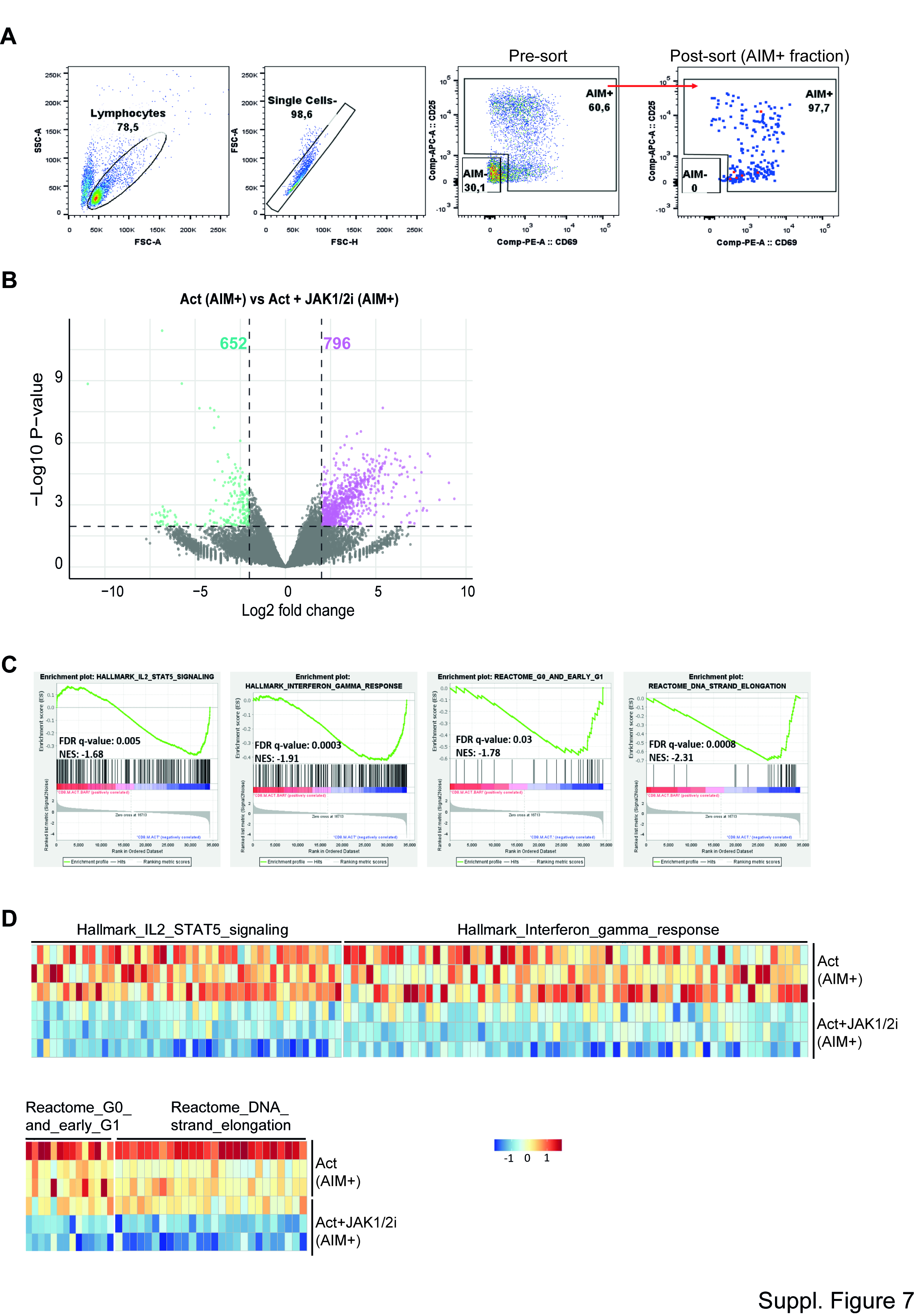

Supplement: Supplementary file 8 — Fig. S7 [file 41419_2026_8610_MOESM8_ESM.tif]

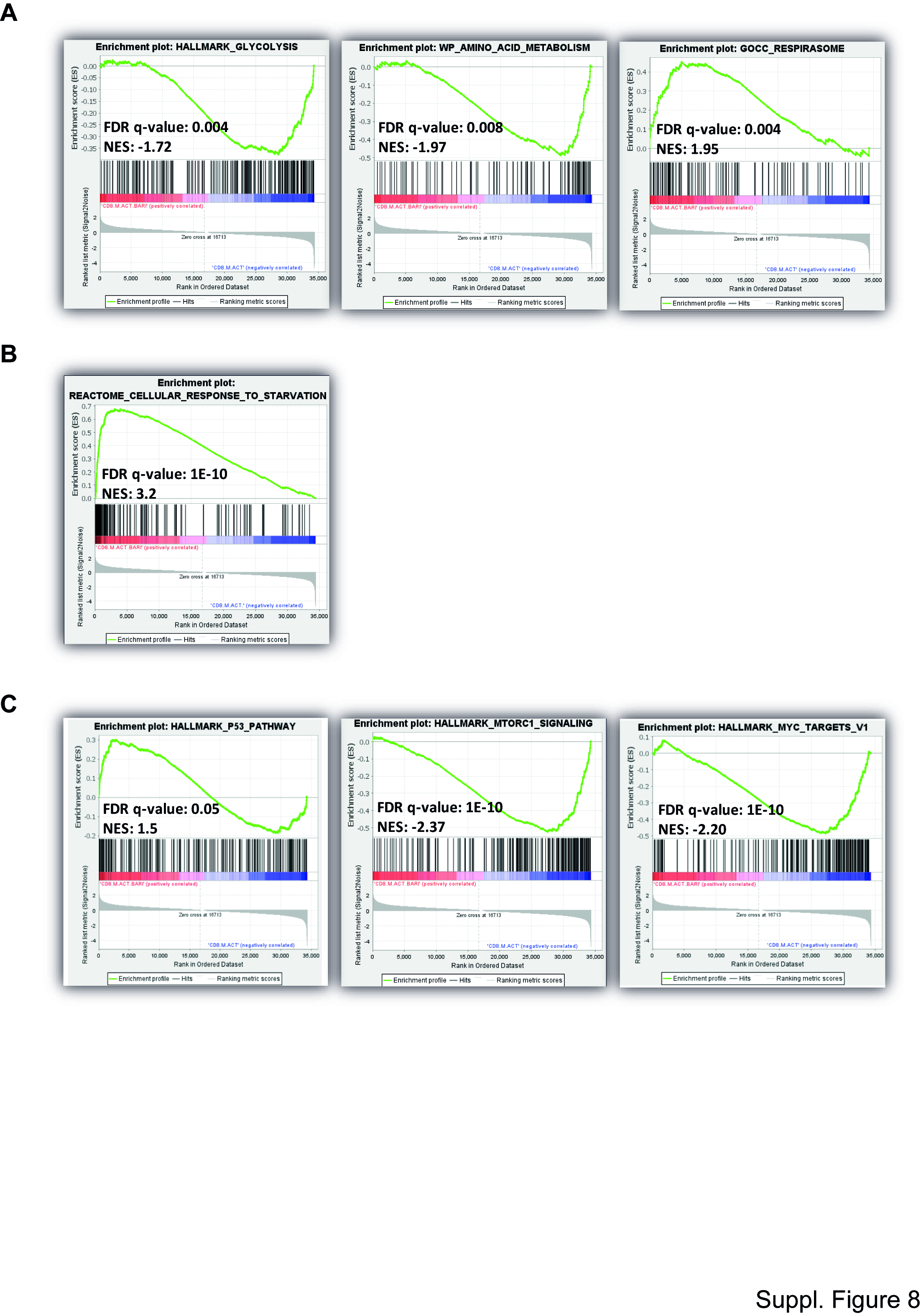

Supplement: Supplementary file 9 — Fig. S8 [file 41419_2026_8610_MOESM9_ESM.tif]

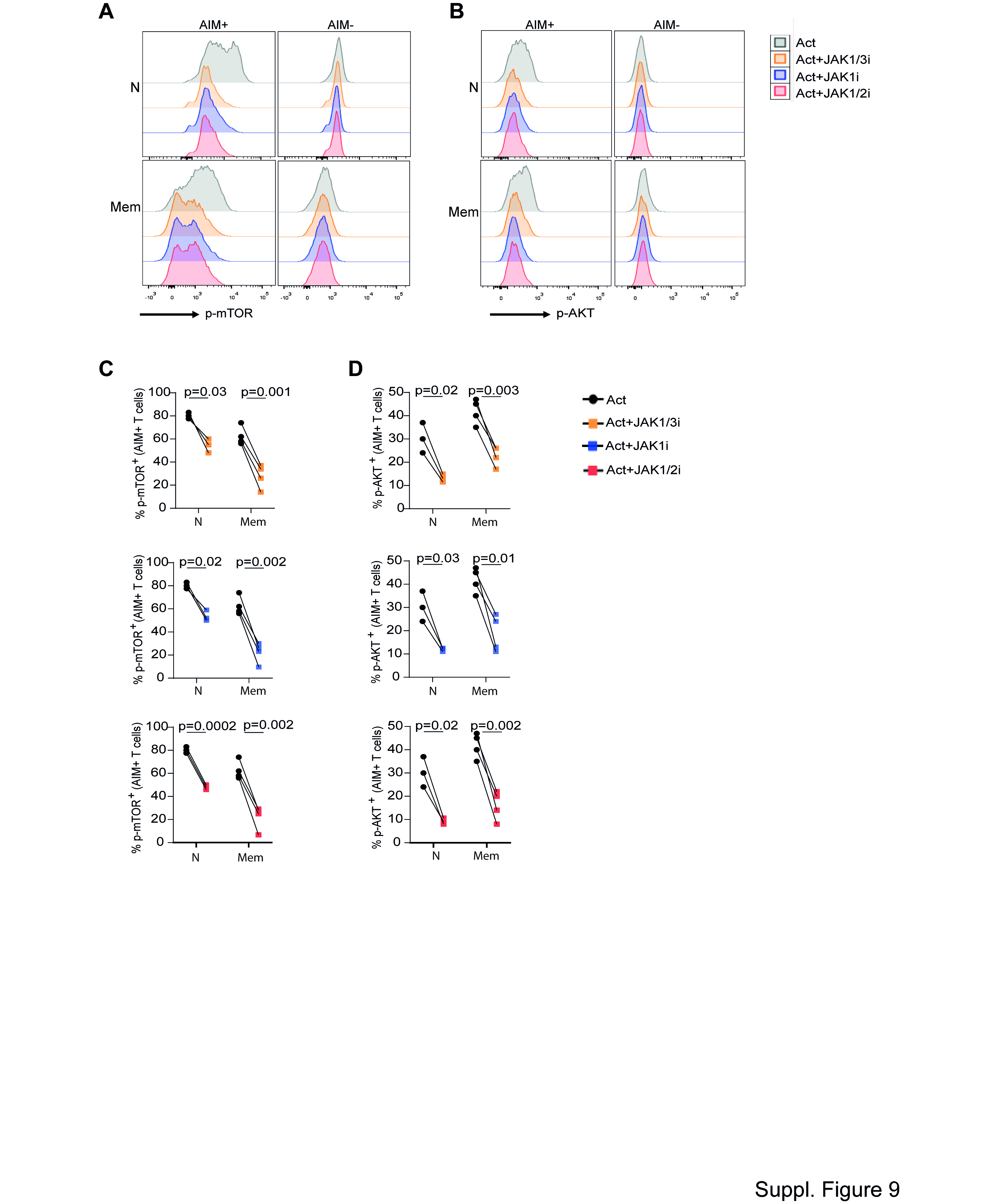

Supplement: Supplementary file 10 — Fig. S9 [file 41419_2026_8610_MOESM10_ESM.tif]
